# Molecular Cloning and Functional Analysis of Holin and Endolysin From *Escherichia* Phage UE‐M6 as Potential Antibacterial Agents

**DOI:** 10.1002/mbo3.70150

**Published:** 2025-11-28

**Authors:** Hira Niaz, Mikael Skurnik, Fazal Adnan

**Affiliations:** ^1^ Atta ur Rahman School of Applied Biosciences (ASAB) National University of Sciences and Technology (NUST) Islamabad Pakistan; ^2^ Department of Bacteriology and Immunology, Human Microbiome Research Program, Faculty of Medicine University of Helsinki Helsinki Finland

**Keywords:** antibacterial activity, antibiotic‐resistant, docking, endolysin, Gram‐negative and Gram‐positive, holin, PhageUE‐M6

## Abstract

The rise of antibiotic‐resistant bacteria has intensified the search for alternative antibacterial strategies. Bacteriophage (phage) therapy is gaining attention as a promising approach, utilizing phage‐derived proteins such as holins and endolysins to combat bacterial infections. In this study, the endolysin (UE‐lysin) and holin (UE‐holin) genes from *Escherichia* phage UE‐M6 were characterized, and their antimicrobial activity was evaluated. In silico analysis revealed that UE‐lysin has a modular architecture, with the *N*‐terminal enzymatic activity domain that contains an *N*‐acetylmuramidase of the glycoside hydrolase family GH108, and the *C*‐terminal cell wall‐binding domain that contains the peptidoglycan binding family PG_binding_3 domain. UE‐holin was predicted to belong to class II holins, featuring two transmembrane helices. Furthermore, the genes encoding the UE‐lysin and UE‐holin were cloned and their expression optimized in *Escherichia coli* BL21 (DE3). The purified recombinant UE‐lysin (27 kDa) and UE‐holin (15 kDa) exhibited antibacterial activity against the *E. coli* host strain PSU‐5266 (UE‐17). The addition of the outer membrane permeabilizer ethylenediaminetetraacetic acid further enhanced their activity. Notably, the combined application of UE‐holin and UE‐lysin demonstrated greater antibacterial efficacy than either enzyme alone, highlighting a synergistic effect. Furthermore, UE‐lysin and UE‐holin exhibited high lytic activity against *E. coli*, *Bacillus*, and *Staphylococcus aureus* strains, underscoring their potential as candidates for treating both Gram‐negative and Gram‐positive bacterial infections.

## Graphical abstract

We isolated phage UE‐M6 from wastewater and subsequently cloned, expressed, and purified its encoded endolysin (UE‐lysin) and holin (UE‐holin). Our results demonstrate that the combined application of UE‐lysin and UE‐holin exhibits significantly greater antibacterial activity than either protein alone, indicating a synergistic interaction between the two. Furthermore, both UE‐lysin and UE‐holin displayed antibacterial activity against a range of Gram‐negative and Gram‐positive bacteria.

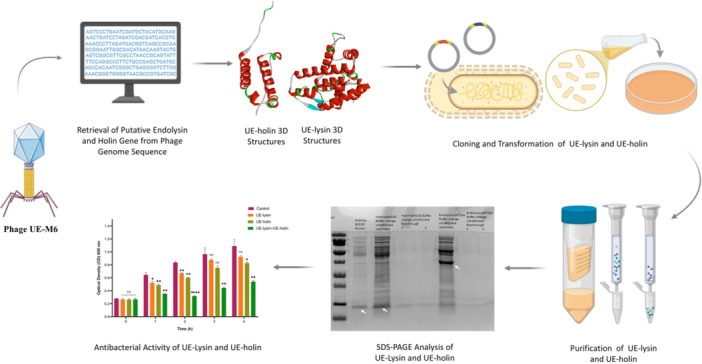

## 1 Introduction

Antimicrobial resistance (AMR) has become a significant global challenge, posing serious threats to public health and development. It is estimated that in 2021, bacterial AMR was directly linked to 4.71 million deaths worldwide, with a forecast of escalation to 8.22 million deaths by 2050 (Naghavi 2024). Beyond its impact on mortality, AMR imposes a severe economic burden. According to World Bank estimates, by 2050, AMR could result in an additional US$1 trillion healthcare cost and annual global gross domestic product (GDP) losses of US$1 trillion to US$3.4 trillion by 2030 (Jonas 2017).

To tackle the increasing AMR threats, researchers are exploring various alternative strategies, with phage therapy emerging as a promising approach. Phage therapy, which involves the use of bacteriophages (viruses that infect bacteria), has been studied for nearly a century as a potential treatment for bacterial infections (Skurnik et al. 2025). However, the use of whole phages in human therapy remains limited in many countries due to concerns regarding dose optimization, the potential development of phage‐resistant bacterial strains, immunogenicity, large‐scale production challenges, and stringent regulatory and legislative barriers (Danis‐Wlodarczyk et al. 2021). To overcome these limitations, increasing attention has been directed toward phage‐derived lytic enzymes. These enzymes, including endolysins and holins, offer several advantages over whole‐phage therapy. They reduce the likelihood of bacterial resistance, minimize unwanted immunological responses, and exhibit broad‐spectrum antibacterial activity (Boroujeni et al. 2024). Several preclinical studies and recent phase I/II clinical trials have demonstrated the safety and efficacy of lysin‐based therapeutics, highlighting their potential for clinical application (Abdelkader et al. 2019).

Endolysins, also called lysins, are a class of peptidoglycan (PG) hydrolases synthesized by double‐stranded DNA (dsDNA) phages. These enzymes play a crucial role in the phage lytic cycle by enzymatically degrading the PG component of the bacterial cell wall, leading to host cell lysis (Rahman et al. 2021). On the basis of their specific cleavage sites in the PG, endolysins are classified into three major categories. Glycosidases and lytic transglycosylases cleave glycosidic bonds between *N*‐acetylmuramic acid (NAM) and *N*‐acetylglucosamine (NAG). Amidases break the amide bond between the glycan moiety (NAM) and the peptide moiety (l‐alanine). Endopeptidases cleave dipeptide bonds within the stem peptide of the PG (Sabur et al. 2025). The varied enzymatic activities of endolysins make them powerful antimicrobial agents, offering significant potential for phage therapy and the development of alternative treatments for bacterial infections. Structural diversity exists among endolysins from Gram‐positive and Gram‐negative bacteria. Most endolysins from Gram‐positive bacteria have a modular structure, are larger, and have a molecular weight of 25–40 kDa. They typically consist of two distinct domains: an *N*‐terminal enzymatically active domain (EAD) and a C‐terminal cell wall‐binding domain (CBD), connected by a short, flexible linker. In contrast, most endolysins from Gram‐negative bacteria are smaller, with a molecular weight ranging from 15 to 20 kDa, and usually possess a single globular EAD domain (Broendum et al. 2018). However, a few Gram‐negative endolysins with a modular structure have been identified, such as Abp013, an endolysin from *Acinetobacter baumannii* bacteriophage φAbp2 (Chu et al. 2022), and the SPN1S endolysin from *Salmonella typhimurium* SPN1S bacteriophage (Park et al. 2014).

Holin is another essential and diverse group of phage‐encoded proteins, primarily found in dsDNA phages. These small, hydrophobic transmembrane (TM) proteins accumulate in the cytoplasmic membrane during the infection cycle. Once they reach a critical threshold in the late stage of infection, holins form pores in the cytoplasmic membrane, allowing endolysins to access and degrade the PG layer of the bacterial cell wall. This coordinated action leads to cell lysis, facilitating the release of newly assembled phage particles (Abeysekera et al. 2022). Holins are small proteins, typically ranging from 50 to 210 amino acids in length, with a molecular weight of approximately 5–20 kDa. On the basis of the number of transmembrane domains (TMDs) and their amino acid sequences, holins are classified into three main classes (Wang et al. 2000). Class I holins are the largest, containing more than 95 amino acids and 3 TMDs, with the C‐terminus in the cytoplasm and the *N*‐terminus in the periplasm. An example of this class is the hol15 protein from *Staphylococcus aureus* phage P68 (Takáč et al. 2005). Class II holins are smaller, ranging from 65 to 95 amino acids and containing 2 TMDs, with both the C‐terminus and *N*‐terminus in the cytoplasm. A well‐known example is the S protein from the lambdoid phage 21 (Pang et al. 2009). Class III holins are the smallest, consisting of 1 TMD, with the C‐terminus in the periplasm and the *N*‐terminus in the cytoplasm, such as the holins found in phages ФCP39O and ФCP26F (Seal et al. 2011). These structural variations in holins contribute to their diverse roles in phage‐induced bacterial cell lysis

Various studies have been performed on the therapeutic utilization of endolysins (Golban et al. 2025; Jindanuch 2024; Ramesh et al. 2022), yet the research on the coordinated action of holin and endolysin remains limited. Understanding their combined antibacterial efficacy is crucial, particularly against clinically relevant pathogens where antibiotic resistance poses a growing threat.

Therefore, the current study focuses on the molecular cloning and functional characterization of holin and endolysin genes from *Escherichia* phage UE‐M6. By expressing and purifying these enzymes, we investigated their individual and combined antibacterial activities against clinically relevant Gram‐positive and Gram‐negative bacterial strains. We hypothesize that phage‐derived endolysin and holin exhibit significant antibacterial activity and that their synergistic application enhances bactericidal efficacy compared with individual treatments.

## 2 Methods

### 2.1 Bacterial Strains, Phage, Plasmid, and Growth Conditions

The *Escherichia coli* host strain PSU‐5266 (UE‐17), serotype O25:H4, isolated from a human urine sample, and the phage UE‐M6 (accession number PP301343), isolated from wastewater, have been described previously (Niaz et al. 2025). The bacterial strains, plasmids, and primers used in this study are listed in Table 1. All *E. coli* strains were grown in lysogeny broth (LB) medium at 37°C at 150–180 rpm, unless stated otherwise, or on LB supplemented with 1.5% agar (LA). When required, kanamycin (Km) 50 μg/mL or ampicillin (Amp) 100 μg/mL was added to the medium. For T7 promoter induction, 1 mM isopropyl‐β‐d‐thiogalactopyranoside (IPTG) was used, while 0.1% l‐arabinose was added for the *araBAD* promoter induction. All the reagents and kits used in this study are listed in Table S1.

**Table 1:** Bacterial strains, plasmids, and primers used in this study.

	Genotype or relevant properties	Source/Reference
*Bacteria*		
*Escherichia coli* DH5α	*supE*44, *ΔlacU*169(φ80 *lacZ* ΔM15), *hsdR*17, *recA*, *endA*1, *gyrA*96, *thi‐1*, *relA*1	Invitrogen
*E. coli* BL21 (DE3)	F^‐^, *ompT*, *gal*, *dcm*, *lon*,*hsdSB*(rB‐mB‐), *λ*(DE3)	Novagen
*E. coli*, Novablue (DE3)	F, *recA1*, *endA1*, *hsdR17*(rK−, mK+), *supE44*, *thi‐1*, *gyrA96*, *relA1*, λ(DE3)	Novagen
*E. coli* Rosetta (DE3)	F^‐^, *ompT*, *hsdSB (rB‐mB‐) gal dcm (DE3) pRARE (argU*, *argW*, *ilex*, *glyT*, *leuW*, *proL) (CamR)*	Novagen
*Plasmids*		
pET28α	KanR; expression vector with His‐Tag	Novagen
pET28α‐lysin	The UE‐lysin gene cloned into pET28α	This study
pET28α‐holin	The UE‐holin gene cloned into pET28α	This study
pBAD30	AmpR; expression vector	Guzman et al. (1995)
pBAD30‐holin	The UE‐holin gene cloned into pBAD30	This study
*Primers* a		
UE‐lysin F	AGCCTGGGATCCATGAAAGCTAAACAGAAACTTGCTGCAAA	This study
UE‐lysin R	AGCGCTGAAGCTTAGAGATTAGCATCTTCATTACATGCCTCCAG	This study
UE‐holin F	AGCCTGGGATCCATGGTTCGCAGGCTAAAAAGAAAAGTAGAG	This study
UE‐holin R	AGCGCTGAAGCTTAGCTTTCATGTGAATCTTCCTTTGGTCG	This study
pET28α F	CCCCTATAGTGAGTCGTATTAA	Skurnik Lab
pET28α R	CTAGTTATTGCTCAGCGGT	Skurnik Lab

a Restriction sites are underlined.

### 2.2 Bioinformatic Analysis

The amino acid sequences of UE‐lysin (WWE95589.1) and UE‐holin (WWE95588.1) were used for homology search in the NCBI database via the BLASTp server (https://blast.ncbi.nlm.nih.gov/Blast.cgi). The physicochemical properties of UE‐lysin and UE‐holin were assessed using the ExPASy‐Protparam tool (https://web.expasy.org/cgi-bin/protparam/protparam). Conserved domain analysis was performed through InterProScan (Blum 2025) and BLASTp tools. TM helicase and signal peptides were predicted via DeepTMHMM (Hallgren 2022) and signal 6.0 (Nielsen 2017), respectively. The three‐dimensional (3D) structure of proteins was modeled using the trRosetta webserver using default parameters (Du et al. 2021). The quality of the predicted structures was assessed using SAVES Procheck (https://saves.mbi.ucla.edu/) with ERRAT‐quality scores and Ramachandran scores.

### 2.3 Phage DNA Extraction

Genomic DNA was extracted using phenol chloroform extraction method as described previously (Sambrook and Russell 2006). Briefly, 400 µL phage lysate was added to a 1.5‐mL Eppendorf, then 1.3 µL DNase I (1 U/µL) and 4 µL RNase A (1 mg/mL) were added and incubated at 37°C for 30 min. Subsequently, 16 µL ethylenediaminetetraacetic acid (EDTA) (0.5 M), 1.2 µL Protease K (20 mg/mL), and 20 µL sodium dodecyl sulfate (SDS) (10%) were added, followed by incubation at 56°C for 60 min. After cooling to room temperature, 400 µL (1 vol) of phenol was added, mixed gently by turning the tube for 15 min then centrifuged at 14k rpm for 5 min. The aqueous phase was collected, and extraction was repeated until no interface precipitate remained. A final extraction with 1 vol chloroform was performed to remove residual phenol. DNA was precipitated by adding 0.1 vol sodium acetate (3 M, pH 7.0) and 2 vol absolute ethanol, and the tubes were inverted until DNA threads became visible. DNA was washed with 1 mL 70% ethanol, centrifuged for 15 min at 13k rpm, air dried for 10 mints and dissolved in 50 µL tris‐EDTA (TE) buffer. Samples were incubated overnight for complete dissolvement. DNA concentration was measured by Qubit Fluorometer device (Invitrogen, Thermo Fisher Scientific).

### 2.4 Polymerase Chain Reaction (PCR) Amplification and Cloning of Phage Genes into Recombinant Plasmids

Genomic DNA of Phage UE‐M6 was used as template to amplify the UE‐holin and UE‐endolysin genes with the primers mentioned in Table 1, using high‐fidelity DNA polymerase and the T‐Gradient thermal cycler. PCR conditions consisted of initial denaturation at 95°C for 5 min, followed by 30 cycles of denaturation at 95°C for 20 s, annealing at 55.7°C for 30 s, extension at 72°C for 60 s, with a final extension step at 72°C for 5 min. The resulting PCR products were purified by PCR purification kit (Sangon Biotech, Shanghai, China) following the manufacturer's instructions. The purified PCR products were then digested with HindIII and BamHI in fast digest buffer (Thermo Fisher Scientific). The digested endolysin and holin gene fragments were ligated into pET28α, creating recombinant plasmids pET28α‐lysin and pET28α‐holin. The holin gene was also ligated into pBAD30 to generate plasmid pBAD30‐holin. The recombinant plasmids were transformed into *E. coli* strain DH5α using the heat shock method (Froger and Hall 2007). Transformed colonies were selected on LA plates supplemented with kanamycin (50 μg/mL) or ampicillin (100 μg/mL). The inserted genes were further confirmed by Sanger sequencing (FIMM Technology Centre, Finland). The resulting chromatograms and FASTA files were analyzed using Staden Package programs (Staden 1996) to visualize chromatograms and align sequences against the reference genes.

### 2.5 Protein Expression and Purification of UE‐Holin and UE‐Lysin

For protein expression analysis, the constructed plasmids pET28α‐lysin, pET28α‐holin, and pBAD30‐holin plasmids were transformed into competent *E. coli* BL21(DE3), Novablue (DE3), and Rosetta (DE3) bacteria. Transformed colonies were inoculated into LB supplemented with kanamycin (50 μg/mL) or ampicillin (100 μg/mL) and incubated shaking (150 rpm) overnight at 37°C. A 1% inoculum from the overnight culture was transferred into fresh LB and allowed to grow until OD_600_ reached 0.7. Protein expression from pET28α‐lysin and pET28α‐holin was induced with 1 mM IPTG, while pBAD30‐holin expression was induced with 0.1% l‐arabinose. Cultures were then incubated at 16°C for an additional 16–18 h.

Cells were harvested by centrifugation at 5000*g* for 30 min, the supernatant was discarded, and the pellet was resuspended in lysis buffer (0.5 M NaCl, 20 mM NaH_2_PO_4_, and pH 7.2). Cell lysis was performed using a rapid freeze–thaw method, followed by treatment with DNase, RNase, lysozyme, and protease inhibitor tablets (Pierce Protease Inhibitor mini‐Tablets, EDTA‐free, Thermo Fisher Scientific) (Gomez‐Raya‐Vilanova et al. 2022). Sonication was conducted using a Branson sonifier (Branson Ultrasonics, Danbury, USA) with a 30% duty cycle and an output control setting of 2. Samples were sonicated for 10 cycles of 30 s, with 30‐s pauses between each cycle. The lysate was centrifuged at 10,000*g* at 4°C for 30 min, and the supernatant was decanted while the pellet was resuspended in lysis buffer. The proteins from the supernatant (cell lysate) were further purified using Ni‐NTA affinity column chromatography (Qiagen) with protein‐dependent imidazole concentrations. The supernatant (cell lysate) was mixed with 2 mL of Ni‐NTA agarose and incubated with shaking at 4°C for 1 h. The unbound fraction was removed by centrifugation at 1000*g* for 10 s at 4°C. The agarose resin was then washed four times with 10 mL of washing buffer (0.5 M NaCl, 20 mM NaH₂PO₄, and pH 7.2). Bound proteins were eluted in four steps using 1 mL elution buffer (0.5 M NaCl, 20 mM NaH₂PO₄, 50 mM imidazole, and pH 7.2). The unbound supernatant, the first and last wash, and all four elution fractions were analyzed by 12% sodium dodecyl sulfate–polyacrylamide gel electrophoresis (SDS‐PAGE). The protein solutions were ultrafiltrated using a cellulose membrane (Amicon Ultra MWCO 30000 Ultra filtration device, Millipore) to remove imidazole and salt ions. The purity of the protein was assessed via 12% SDS–PAGE, and the protein concentrations were measured using the NanoDrop 2000 Spectrophotometer.

### 2.6 Intracellular Lysis Activity of UE‐Holin and UE‐Lysin

The effect of UE‐lysin and UE‐holin on the growth of *E. coli* BL21 expression host was evaluated by monitoring the OD_600_ values of the cultures over time (Zhang et al. 2022). Briefly, the *E. coli* BL21 strain harboring the plasmid pET28α‐lysin or pBAD30‐holin was grown until OD_600_ reached 0.6. The induction was then carried out using 2 mM IPTG for the pET28α‐lysin strains, and 0.2% l‐arabinose for the pBAD30‐holin strain. Cultures were grown at 37°C, and OD_600_ values were measured for 3 h at half‐hour intervals. The uninduced strains served as controls, and the assays were performed in triplicate.

### 2.7 Antibacterial Activity of UE‐Holin and UE‐Lysin Using Turbidity Assay

The antibacterial activities of the UE‐holin and UE‐lysin were tested on the *E. coli* strain PSU‐5266 (UE‐17). Since Gram‐negative bacteria have an outer membrane that can hinder enzyme activity, EDTA was used as a permeabilizer (Yin et al. 2022).

To determine the appropriate EDTA concentrations, PSU‐5266 (UE‐17) was grown in LB at 37°C with shaking (150 rpm) for 3–4 h until reaching an OD₆₀₀ of 0.6. Fifty µL aliquots of EDTA solutions (10, 5, 2, and 1 mM) were mixed with 100 µL of the bacterial suspension, and the OD₆₀₀ values of the cultures were measured over 4 h.

100 µL of the bacterial suspension (OD₆₀₀ ≈ 0.6), 100 µL of the purified enzyme (UE‐holin [2.57 mg/mL] or UE‐lysin [1.47 mg/mL]), and 50 µL of the optimal EDTA concentration were combined in a 96‐well microtiter plate. The plate was incubated at 37°C with constant shaking, and OD₆₀₀ values were recorded every hour for 4 h. Controls included bacteria alone and bacteria with EDTA. Activity of UE‐lysin and UE‐holin activity was also assessed without EDTA following the same protocol.

### 2.8 Antibacterial Activity of UE‐holin and UE‐lysin Using Well Diffusion Method

The antibacterial activities of the UE‐lysin and UE‐holin were further validated by the well diffusion method, with the presence and absence of EDTA (Ramesh et al. 2022). Overnight colonies of PSU‐5266 (UE‐17) were used to prepared 0.5 MacFarland bacterial suspension. Then, 100 µL of this suspension was evenly spread on Mueller–Hinton agar plates (MHA) to prepare bacterial lawns. Four wells of 6 mm in diameter were pierced on each plate, and 100 µL of UE‐lysin (2.57 mg/mL) and/or UE‐holin (1.47 mg/mL) was added to each well. Cell lysate of *E. coli* BL21 harboring pET28α was used as a negative control.

### 2.9 Determination of Broad‐Spectrum Activity

The antibacterial spectrum of UE‐lysin and UE‐holin was evaluated against 10 bacterial strains (Table S2), including six pathogenic *E. coli* strains (PSU‐5265, UE‐87, STEC, CME‐5, PE‐127, and FE‐28), three *S. aureus* strains (G‐19, G‐21, and G‐LR), and one *Bacillus safensis* strain (*Bacillus* 4B). The Gram‐negative strains were permeabilized following a previously described method (Khakhum et al. 2016). Briefly, bacterial cultures were grown in LB to the exponential phase (OD_600_ ~ 0.5–0.6), harvested by centrifugation at 4k rpm for 15 min at 4°C. The collected pellets were permeabilized using chloroform‐saturated 0.05 M Tris–HCl buffer, pH 7. The samples were shaken at room temperature for 45 min, after which the permeabilized cells were washed, resuspended in phosphate buffer (10 mM and pH 8.0) and adjusted to an OD_600_ of 0.6–0.8. Subsequently, 180 µL of the bacterial suspension was transferred into a 96‐well microtiter plate, followed by the addition of 20 µL of either UE‐holin or UE‐lysin. Bacterial cultures without enzyme treatment served as negative controls, whereas cultures treated with enzymes were used as experimental samples.

### 2.10 Statistical Analysis

All statistical analyses were performed using GraphPad Prism software (version 8.0.1). All experiments were conducted in biological triplicates and results are presented as mean values with standard deviation (SD), with error bars representing the variation across experiments. Statistical significance was assessed using two‐way analysis of variance using Dunnett's multiple comparison test. A *p* < 0.005 was considered statistically significant.

## 3 Results

### 3.1 Sequence Analysis of UE‐Lysin and UE‐Holin

The 76,110 bp genome of phage UE‐M6 contains 88 predicted genes among which genes *14* and *15* were predicted to encode the holin and endolysin, respectively (Niaz et al. 2025). These lysis‐associated genes are arranged in a canonical order, with the holin encoding gene *14* (13,276–13,536) located upstream of the endolysin encoding gene *15* (13,526–14,161).

The holin protein, designated as UE‐holin, consists of 86 amino acids with a calculated molecular weight of 10 kDa and an isoelectric point of 10.50. BLASTp analysis showed that the UE‐holin shares 96.51% and 94.19% sequence similarity with the *Escherichia* phage vB_EcoS_Uz‐1 (UWJ04308.1) and the Shigella phage pSb‐1 (YP_009008416.1) holins, respectively. Deep TMHMM analysis predicted two TM helices (aa residues 22–37 and 61–71), a cytoplasmic *N*‐terminus (aa residues 1–21), and a charged C‐terminus (aa residues 72–86), indicating that the UE‐holin likely belongs to class II holins (Figure S1).

The endolysin protein, designated as UE‐lysin, consists of 211 amino acids with a molecular weight of 23 kDa and an isoelectric point of 9.05. BLASTp analysis indicated 99.05% and 98.10% similarities to *Shigella* phage pSb‐1 (YP_009008415.1) and *Escherichia* phage Bp4 (YP_009031972.2) endolysins, respectively. Domain analysis predicted a modular architecture: the *N*‐terminal enzymatic activity domain (EAD) contains the _glycoside hydrolase family GH108 *N*‐acetylmuramidase (PF05838), while the C‐terminal cell CBD contains PG_binding_3 (PF09374) (Figure S2).

**Figure 1:**
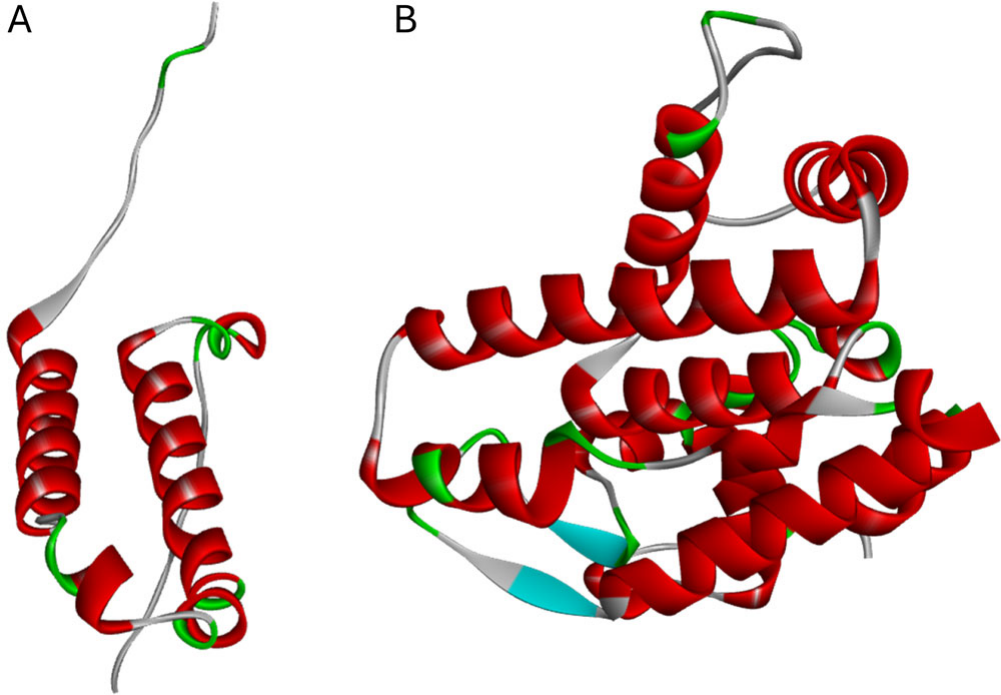
Predicted three‐dimensional structures: (A) UE‐holin (B) UE‐lysin. The protein structures are composed of alpha helices, beta sheets, loops, and turns, and these are represented by red, green, and gray colors, respectively.

The predicted 3D structures of UE‐lysin and UE‐holin are represented in a ribbon model (Figure 1). The overall ERRAT‐quality scores of 100 and Ramachandran scores above 90% indicate that both protein models are high‐quality structures (Figure S3).

### 3.2 PCR Amplification and Cloning

**Figure 2:**
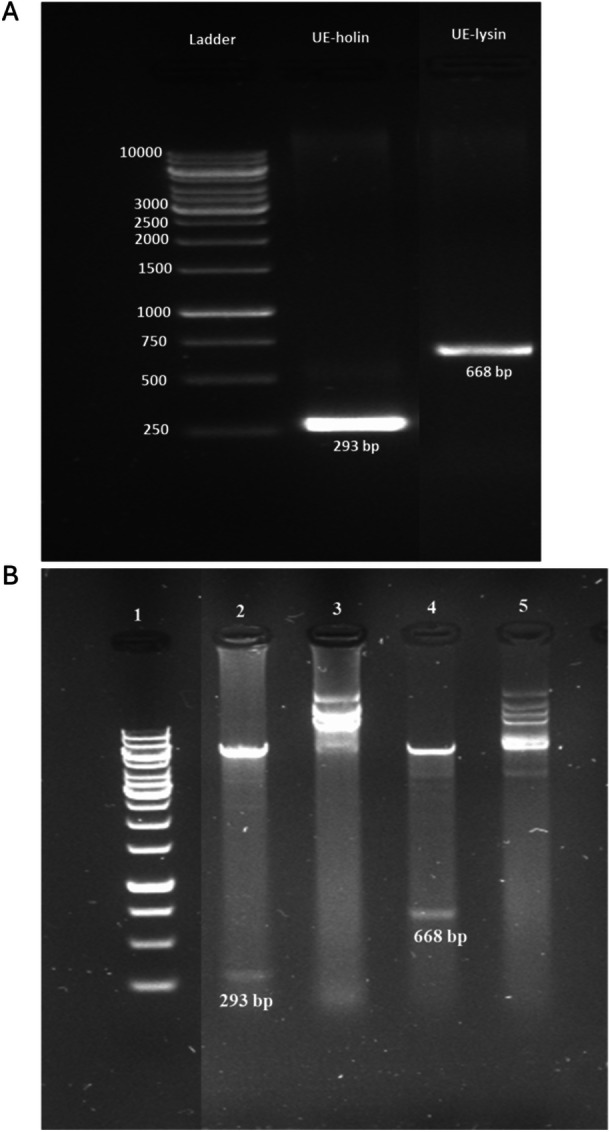
Cloning of UE‐holin and UE‐lysin genes from the UE‐M6 phage. (A) Polymerase chain reaction amplification of UE‐lysin (668 bp) and UE‐holin (293 bp) genes using insert‐specific primers. (B) Restriction digestion analysis of recombinant plasmids using HindIII and BamHI, The digested products generated fragments of the expected size. Lane 1, 1 kb DNA marker; lane 2, pET28α‐holin; lanes 3 and 5, undigested control; lane 4, pET28α‐lysin.

The UE‐lysin and UE‐holin genes of phage UE‐M6 were amplified by PCR using the phage DNA as template and gene‐specific primers (Table 1), yielding PCR products of expected sizes (Figure 2A). The purified amplicons were cloned into pET28α expression vector with cloning success rates of 86% for pET28α‐holin and 75% for pET28α‐lysin. The correct insertions of the target genes were initially confirmed by HindIII/BamHI restriction digestion, which yielded fragments of the expected sizes (Figure 2B). To further validate the constructs, Sanger sequencing was performed, and sequence alignment revealed 100% identity with the reference sequences (Figure S4), confirming the accuracy of the cloning process.

### 3.3 Protein Expression and Purification of UE‐Holin and UE‐Lysin

**Figure 3:**
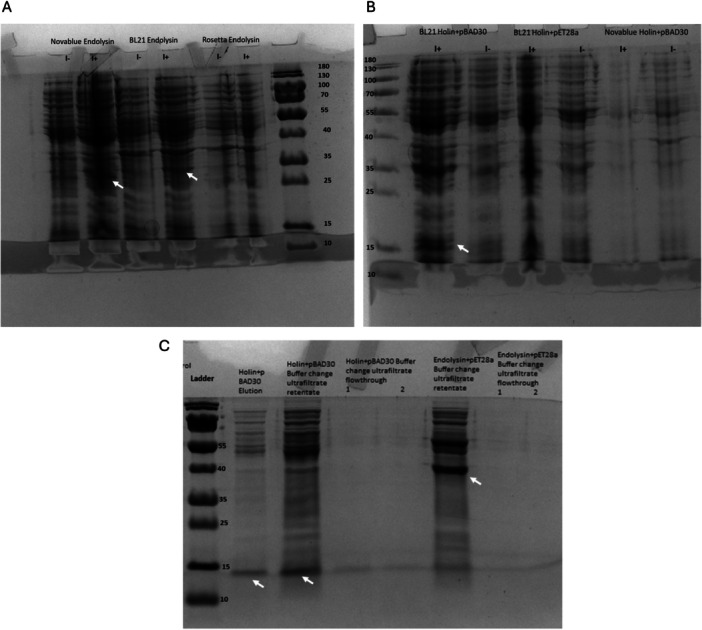
SDS‐PAGE analysis of UE‐Lysin and UE‐holin. (A) Expression of pET28α‐lysin in *Escherichia coli* Novablue, BL21, and Rosetta strains (I^+^, induced; I^‐^, uninduced). (B) Expression of pBAD30‐holin and pET28α‐holin in *E. coli* BL21 and Novablue strains. (C) Concentrated sample of UE‐holin and UE‐lysin (Post Amicon‐15, Millipore). Expressed protein bands are indicated with arrows in all gels. SDS‐PAGE analysis of uninduced and vector‐only control samples is shown in Figures S5 and S6. SDS‐PAGE, sodium dodecyl sulfate–polyacrylamide gel electrophoresis.

To determine a suitable expression host, UE‐lysin and UE‐holin were expressed in *E. coli* BL21(DE3), Novablue(DE3), and Rosetta(DE3) strains. SDS‐PAGE analysis of UE‐lysin revealed a band around 27 kDa in *E. coli* BL21(DE3) and Novablue(DE3) strains (Figure 3A), which closely corresponds to its theoretical molecular weight of 23 kDa. On the basis of these results, *E. coli* BL21(DE3) was selected for large‐scale production. UE‐lysin was then purified using a Ni‐NTA column and ultrafiltration. Interestingly, SDS‐PAGE analysis showed a band around 40 kDa, suggesting a possible dimerization of UE‐lysin (Figures 3C and S5). The final protein concentration was measured at 2.57 mg/mL.

For UE‐holin, expression was observed in the pBAD30‐holin construct within *E. coli* BL21 (DE3), with a band appearing near 15 kDa on SDS‐PAGE (Figure 3B), corresponding to a theoretical 10 kDa weight. Further concentrated protein results confirmed a similar band size for UE‐holin (Figures 3C and S6). The final protein concentration was determined to be 1.47 mg/mL.

### 3.4 Intracellular Lysis Activity of UE‐Holin and UE‐Lysin

**Figure 4:**
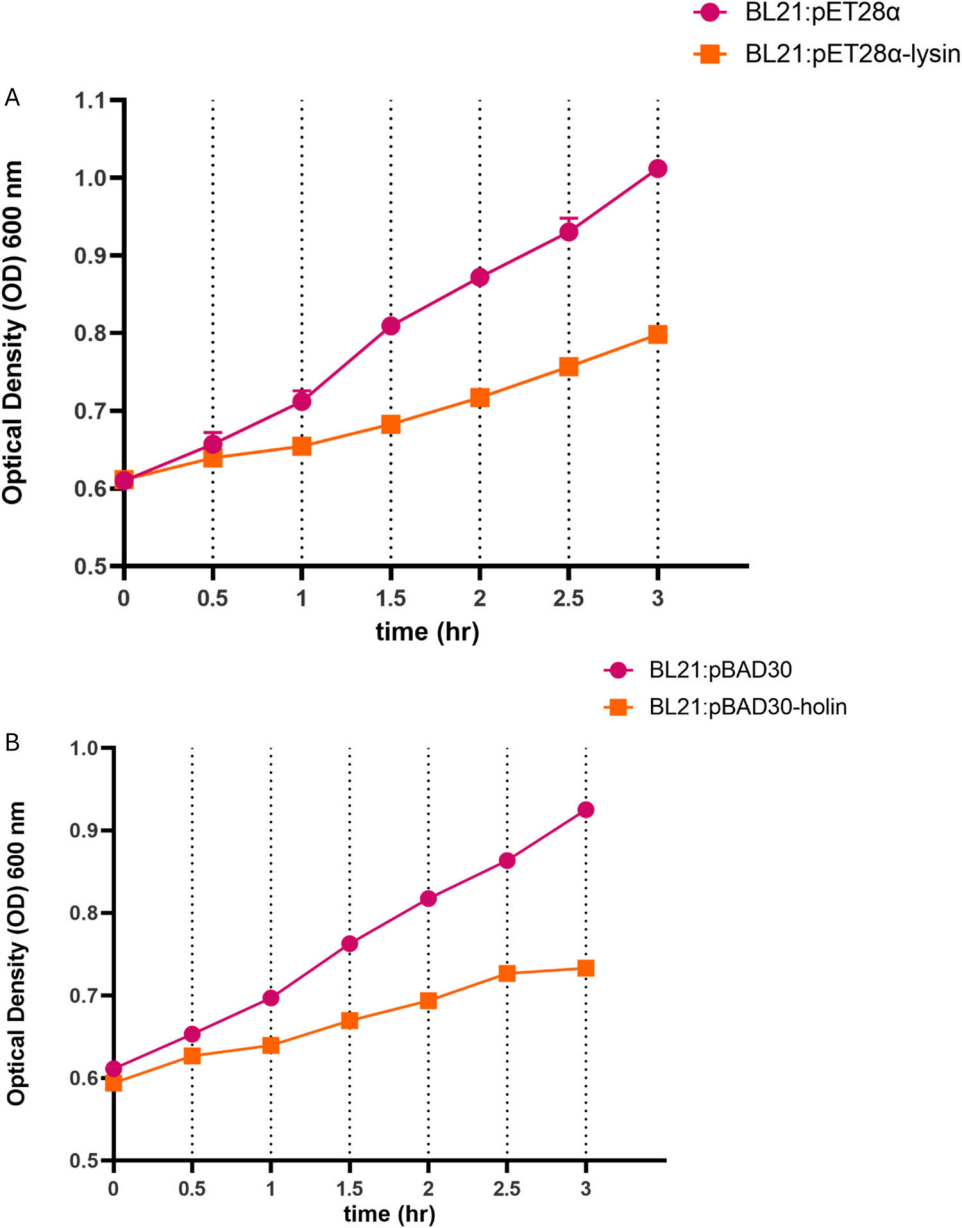
Intracellular lytic activity of UE‐lysin and UE‐holin. (A) 2 mM isopropyl‐β‐d‐thiogalactopyranoside was used to induce the expression of UE‐lysin in *Escherichia coli* BL21, and the strain carrying the empty pET28α vector was used as a control. (B) 0.2% l‐arabinose was used to induce the expression of UE‐holin in *E. coli* BL21, the strain carrying the empty pBAD30 vector was used as a control.

The intracellular lysis activity of UE‐holin and UE‐lysin on the *E. coli* BL21 strain was assessed by measuring the change in OD_600_ value over a 3‐h period following induction. Once induced, *E. coli* BL21 harboring the pET28α‐lysin demonstrated a significant reduction in growth compared with the control strain carrying the empty pET28α vector. After 3 h, the OD_600_ of the control culture reached approximately 1.0, whereas the OD_600_ of the UE‐lysin expressing culture was significantly lower at 0.8 (*p* < 0.0001), indicating a growth inhibitory effect of UE‐lysin (Figure 4A).

A comparable reduction in growth was observed in *E. coli* BL21 expressing UE‐holin. Following 3 h of induction, the OD_600_ of the UE‐holin expressing culture was approximately 0.7, significantly lower than the control culture, which reached an OD_600_ of 0.9 (*p* < 0.0001) (Figure 4B). These findings suggest that both UE‐lysin and UE‐holin exhibit intracellular lytic activity, leading to impaired bacterial growth.

### 3.5 Lytic Activity of UE‐Holin and UE‐Lysin Using Turbidity Assay

The effect of EDTA concentration on bacterial cells was initially assessed to determine the optimal condition for enhancing enzyme activity. The results (Figure S7) demonstrated that at 10 mM EDTA, bacterial growth was significantly inhibited, as indicated by a lower OD_600_ compared with the control. However, at 5, 2, and 1 mM, bacterial growth remained comparable to the control. On the basis of these observations, 1 mM EDTA was selected for further experiments.

**Figure 5:**
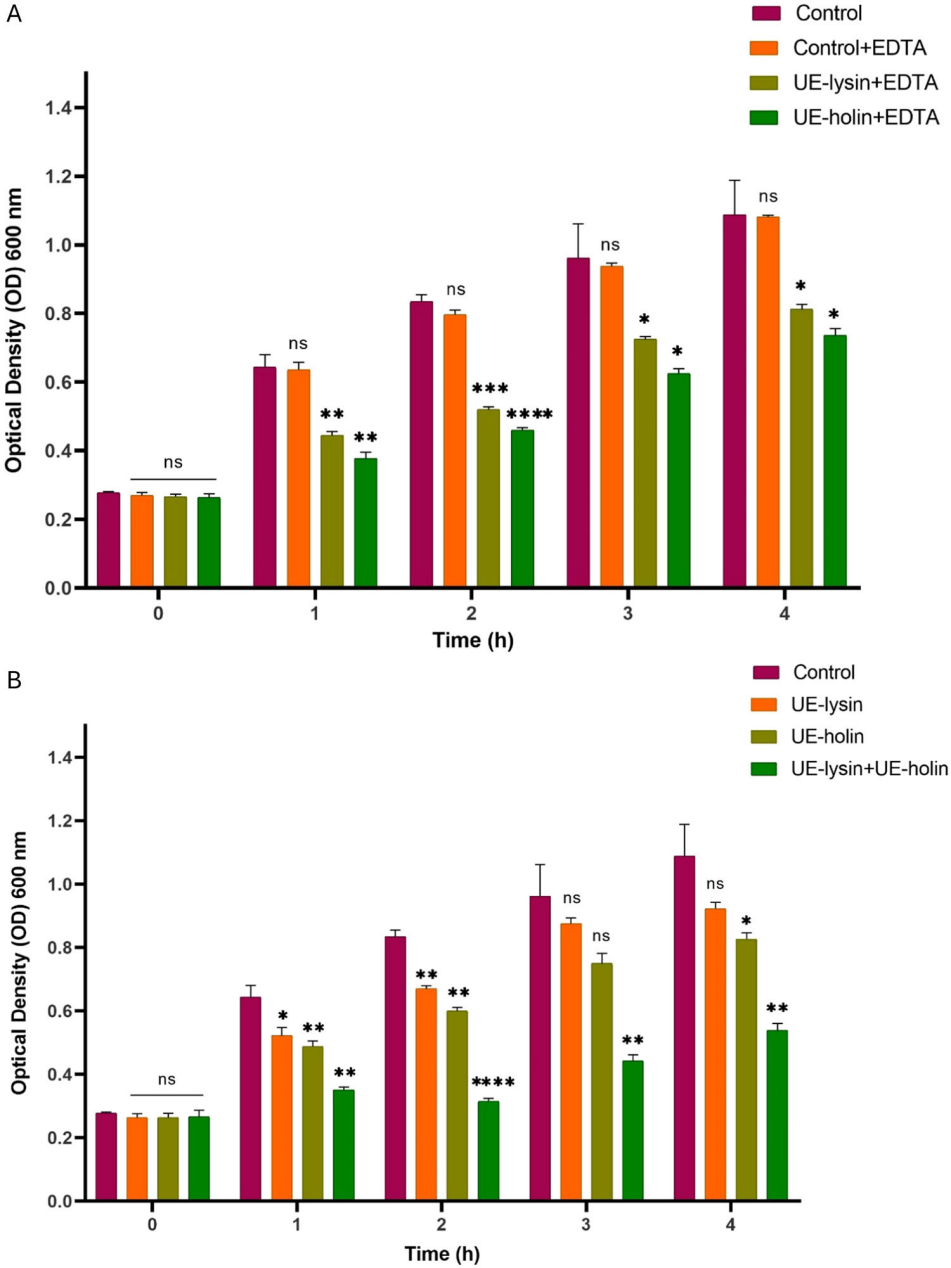
Antibacterial activity of UE‐lysin and UE‐holin measured via turbidity reduction assay (A) with 1 mM EDTA and (B) without EDTA. OD_600_ was measured every hour for 4 h. Data represent the mean values with SD indicated by error bars from three independent experiments. Asterisks indicate levels of statistical significance: *p < 0.05; **p < 0.01; ***p < 0.001; ****p < 0.0001; ns, not significant. EDTA, ethylenediaminetetraacetic acid.

To evaluate the antibacterial activity of UE‐lysin and UE‐holin in combination with EDTA, bacterial growth was monitored by measuring OD_600_ over a 4‐h period (Figure 5A). Protein extract from *E. coli* BL21 harboring pET28α, both with and without EDTA, was used as a negative control in the well diffusion assay and showed no antimicrobial activity. Therefore, in this experiment, the control groups consisted of PSU‐5266 (UE‐17) bacteria alone and bacteria treated with EDTA. Results indicated that the control groups exhibited a steady increase in OD_600_, reaching approximately 1.0 after 4 h of incubation. In contrast, addition of the UE‐lysin (2.57 mg/mL) with EDTA significantly reduced bacterial growth, with OD_600_ reaching only 0.5, compared with 0.8 in the controls after 2 h of incubation (*p* < 0.001). This inhibitory effect persisted until the end of the 4‐h incubation period (*p* < 0.05). UE‐holin (1.47 mg/mL) with EDTA exhibited even greater antibacterial activity, reducing OD_600_ to 0.46 after 2 h (*p* < 0.0001), and this inhibitory effect remained significant throughout the experiment (*p* < 0.05).

The antibacterial activity of UE‐lysin and UE‐holin without EDTA was also assessed (Figure 5B). UE‐lysin alone significantly inhibited bacterial growth during the first 2 h (*p* < 0.01); however, beyond this time point, the reduction in OD_600_ was no longer significant, with OD_600_ stabilizing at 0.9, compared with the control 1.0. On the other hand, UE‐holin alone exhibited a more pronounced effect, causing a significant reduction in OD_600_ within the first 2 h (*p* < 0.001), and this effect remained significant until 4 h (*p* < 0.05). Notably, the combination of UE‐lysin and UE‐holin resulted in a markedly greater antibacterial effect than either enzyme alone, leading to a continuous decline in OD_600_ over the entire 4‐h period (*p* < 0.01). This finding strongly suggests that UE‐lysin and UE‐holin act synergistically, enhancing bacterial lysis when used together.

### 3.6 Antibacterial Activity of UE‐Holin and UE‐Lysin Using Well Diffusion Method

Antibacterial activities of UE‐holin and UE‐lysin were further ratified on MHA plates via the well diffusion method. Cell lysate of *E. coli* BL21 harboring pET28α was served as a negative control.

**Figure 6:**
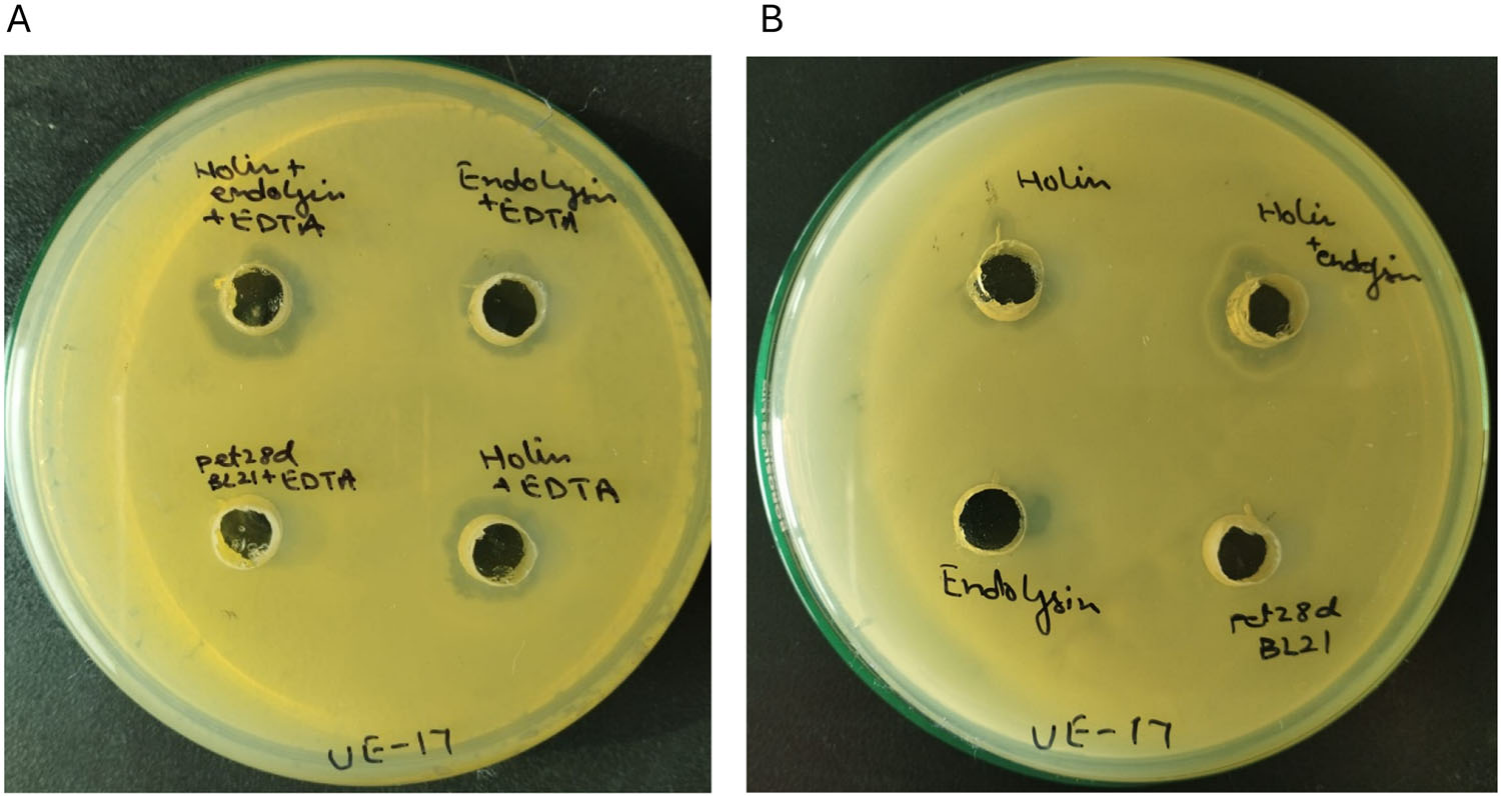
Antibacterial activity of UE‐lysin and UE‐holin measured via the well diffusion method. (A) With 1 mM EDTA and (B) without EDTA, the test was performed against the PSU‐5266 (UE‐17) host strain. The wells labeled with pET28a BL21 (with and without EDTA) contain protein extract from vector‐only bacteria served as negative controls. EDTA, ethylenediaminetetraacetic acid.

Individually, both UE‐holin and UE‐lysin, when combined with 1 mM EDTA, produced clear growth inhibition zones. However, the combination of UE‐holin and UE‐lysin with 1 mM EDTA resulted in a well‐defined and significantly larger zone of inhibition, indicating strong antibacterial activity. Notably, the inhibition was most pronounced when UE‐holin, UE‐lysin, and EDTA were combined, demonstrating an enhanced effect (Figure 6A).

In the absence of EDTA, UE‐holin and UE‐lysin individually exhibited only minimal antibacterial activity, forming significantly smaller zones of clearance. However, when UE‐lysin and UE‐holin are used in combination, they produce a significant clear zone of inhibition (Figure 6B). These findings are consistent with the turbidity reduction assay, further confirming the synergistic effect of UE‐holin and UE‐lysin.

### 3.7 Antibacterial Spectrum of UE‐Lysin and UE‐Holin

**Figure 7:**
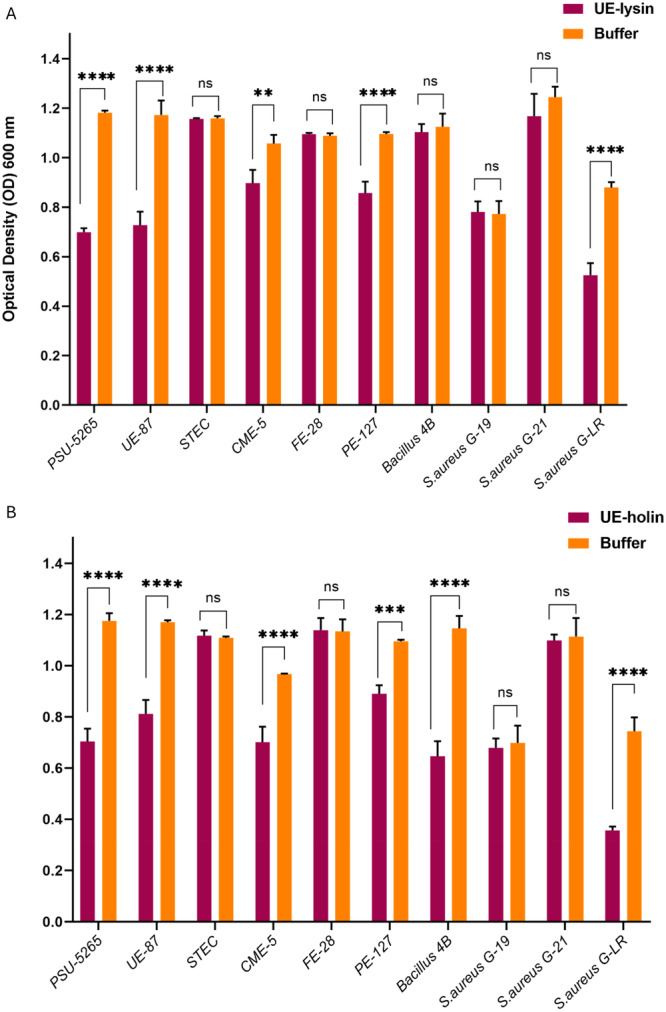
Lytic spectrum of (A) UE‐lysin and (B) UE‐holin against 10 bacterial strains. Bacterial strains were incubated with enzymes for 4 h and their OD_600_ was measured to assess bacterial lysis. Data represent the mean ± standard deviation (SD) from three independent experiments. Asterisks indicate levels of statistical significance: **p < 0.01; ***p < 0.001; ****p < 0.0001; ns, not significant.

The lytic spectrum of UE‐lysin and UE‐holin was evaluated against 10 bacterial strains by measuring OD_600_ after 4 h of incubation. UE‐lysin exhibited a moderate lytic spectrum, effectively lysing 50% of the tested strains (Figure 7a). It significantly reduced OD_600_ in four *E. coli* strains, that is, PSU‐5265, UE‐87, CME‐5, and PE‐127, compared with the control buffer (*p* < 0.0001). Additionally, UE‐lysin demonstrated activity against the Gram‐positive *S. aureus* G‐LR strain, also showing a significant reduction in OD_600_ (*p* < 0.0001).

In comparison, UE‐holin exhibited a broader host range, effectively lysing 60% of the tested strains (Figure 7b). It significantly reduced OD_600_ in the same four *E. coli* strains, that is, PSU‐5265, UE‐87, CME‐5, and PE‐127 (*p* < 0.0001). Moreover, UE‐holin displayed activity against two Gram‐positive strains, that is, *Bacillus* 4B and *S. aureus* G‐LR, with a significant reduction in OD_600_ compared with the control (*p* < 0.0001). These findings indicate that UE‐holin has a broader lytic spectrum than UE‐lysin, making it more effective against both Gram‐negative and Gram‐positive bacteria.

## 4 Discussion

The rise of AMR has intensified the search for alternative antimicrobial strategies. Among these, bacteriophage (phage) therapy or phage‐derived proteins are gaining attention as a promising approach to combat microbial infections. In this study, the genes encoding holin and endolysin of *Escherichia* phage UE‐M6 were cloned and expressed and functionally characterized to evaluate their antibacterial potential.

The *Schitoviridae* family phage UE‐M6 with a 73 kb genome was characterized in our previous study (Niaz et al. 2025). The lysis cassette comprises three genes encoding the holin (gene *14*), the endolysin (gene *15*), and the Rz‐like spanin (gene *16*). This gene arrangement is consistent with the typical architecture of lysis cassettes in phages that infect Gram‐negative bacteria. Our research prioritized the holin–endolysin system, which is fundamental to the lysis mechanism and holds significant therapeutic potential (Cahill and Young 2019). Future studies may explore the role of spanins to further elucidate their contribution to bacterial cell lysis. The holin, designated as UE‐holin, is a small protein that consists of 86 amino acids with a calculated molecular weight of 10 kDa. In silico analysis predicted that there were two TM helices, with a cytoplasmic *N*‐terminus and a charged C‐terminus, indicating that UE‐holin belongs to class II holins (Smith et al. 1998). No conserved domain was identified in UE‐holin, suggesting that it represents a new member of the holin family. The endolysin, designated as UE‐lysin, is a 211‐amino acid protein with a calculated molecular weight of 23 kDa. Most endolysins targeting Gram‐negative typically have a globular shape with a single EAD (Zheng and Zhang 2024). However, the domain analysis of UE‐lysin predicted a modular architecture, with the *N*‐terminal EAD containing an *N*‐acetylmuramidase family domain GH108 (PF05838), while the C‐terminal CBD contains the PG binding domain PG_binding_3 (PF09374). A similar structural organization has been observed in Abp013 lysin from *A. baumannii* bacteriophage, which possesses a glycoside hydrolase family 108 (GH108) domain at the *N*‐terminal and a PG‐binding domain at the C‐terminal end (Chu et al. 2022).

UE‐holin and UE‐lysin were successfully expressed in *E. coli* BL21 (DE3). SDS‐PAGE analysis revealed a 15‐kDa band for UE‐holin and an approximately 27‐kDa band for UE‐lysin, which closely correspond to their calculated molecular weights of 10 and 23 kDa, respectively. The observed differences in size may be attributed to the presence of His tag fusion and the high isoelectric point, which can influence protein mobility (Niu and Guiltinan 1994). Following Ni‐NTA column purification and ultrafiltration, UE‐lysin exhibited a band around ~45 kDa, significantly exceeding its theoretical molecular weight. This suggests multimerization, a well‐documented phenomenon among endolysins. For example, PlyC, a Streptococcal C1 phage endolysin, forms an octamer in solution, consisting of one catalytic subunit and eight binding subunits (Nelson et al. 2006). Similarly, LysB endolysin from the prophage of *Clostridium botulinum* E3 strain Alaska E43 (Morzywolek et al. 2021), Lys170 endolysin, which targets *Enterococcus faecalis* (Proença et al. 2015) and endolysin CD27L, which targets *Clostridium difficile* (Dunne et al. 2016), have also been reported to form multimers. The observed ~45 kDa band for UE‐lysin could correspond to a trimer (3× monomeric MW) or a stable complex with accessory proteins, though further characterization (e.g., size‐exclusion chromatography or native PAGE) is required to confirm its oligomeric state.

The antibacterial activity of UE‐lysin and UE‐holin was evaluated against the PSU‐5266 (UE‐17) bacterial strain, both in the presence and absence of EDTA. EDTA, a well‐known chelating agent, is commonly used to permeabilize the outer membrane of Gram‐negative bacteria, facilitating the action of antibacterial agents (Gontijo et al. 2021). Antibacterial activity was assessed using both the turbidity reduction assay and the well diffusion method. Results demonstrated that UE‐lysin exhibited high activity in the presence of 1 mM EDTA, whereas its activity was significantly reduced in the absence of EDTA. This observation aligns with the behavior of most other endolysins targeting Gram‐negative bacteria (Walmagh et al. 2013; Bai et al. 2019; Nie et al. 2022). UE‐holin displayed antibacterial activity both with and without EDTA; however, its activity was approximately twofold higher in the presence of EDTA. This finding is particularly noteworthy, as holins typically function independently of EDTA, making this an unusual phenomenon. Furthermore, the combined application of UE‐holin and UE‐lysin exhibited greater antibacterial efficacy than either enzyme alone, indicating the holin–endolysin system is necessary for the complete lysis of host cells. This synergism between holin and endolysin is consistent with findings from previous studies, further supporting their cooperative mechanism of action (Basit et al. 2021; Lu et al. 2020; Li et al. 2021). The antibacterial spectrum of the tested proteins was evaluated against six permeabilized Gram‐negative strains and four Gram‐positive strains. UE‐lysin demonstrated high lytic activity against *E. coli* strains, effectively lysing 4 out of 6 strains, but exhibited lysis against only one *S. aureus* strain. Similarly, previous studies have reported that endolysins derived from Gram‐negative bacteria are generally more effective against Gram‐negative strains (Han et al. 2019). UE‐holin displayed an antibacterial spectrum, successfully lysing four *E. coli* strains, one *S. aureus* strain, and one *Bacillus* strain.

## 5 Conclusions

In conclusion, this study explored the cloning, expression, and functional characterization of UE‐holin and UE‐lysin from *Escherichia* phage UE‐M6. Both proteins exhibited individual antibacterial activity, with significantly enhanced efficacy when used in combination against UPEC strains, indicating a synergistic interaction between holin and endolysin. Furthermore, UE‐holin and UE‐lysin demonstrated effective activity targeting both Gram‐negative and Gram‐positive bacteria. While these findings highlight their potential as promising antimicrobial candidates, further studies involving a broader range of clinical isolates are necessary to comprehensively evaluate their spectrum of activity and therapeutic applicability.

## Author Contributions

**Hira Niaz:** conceptualization, formal analysis, investigation, methodology, data curation, visualization, writing – original draft. **Mikael Skurnik:** conceptualization, methodology, validation, project administration, resources, supervision, writing – review and editing. **Fazal Adnan:** conceptualization, validation, funding acquisition, project administration, resources, supervision, writing – review and editing.

## Ethics Statement

The authors have nothing to report.

## Consent

The authors have nothing to report.

## Conflicts of Interest

The authors declare no conflicts of interest.

## Supporting information

Additional file revised.

## Data Availability

The annotated genome sequence of phage UE‐M6 was deposited in the GenBank database under the accession number PP301343. The amino acid sequences of UE‐lysin and UE‐holin, are available under the accession numbers WWE95589.1 and WWE95588.1, respectively.

## References

[mbo370150-bib-0001] Abdelkader, K. , H. Gerstmans , A. Saafan , T. Dishisha , and Y. Briers . 2019. “The Preclinical and Clinical Progress of Bacteriophages and Their Lytic Enzymes: The Parts Are Easier Than the Whole.” Viruses 11, no. 2: 96. 10.3390/v11020096. 30678377 PMC6409994

[mbo370150-bib-0002] Abeysekera, G. S. , M. J. Love , S. H. Manners , C. Billington , and R. C. J. Dobson . 2022. “Bacteriophage‐Encoded Lethal Membrane Disruptors: Advances in Understanding and Potential Applications.” Frontiers in Microbiology 13: 1044143. 10.3389/fmicb.2022.1044143. 36345304 PMC9636201

[mbo370150-bib-0003] Bai, J. , E. Yang , P. S. Chang , and S. Ryu . 2019. “Preparation and Characterization of Endolysin‐Containing Liposomes and Evaluation of Their Antimicrobial Activities Against Gram‐Negative Bacteria.” Enzyme and Microbial Technology 128: 40–48. 31186109 10.1016/j.enzmictec.2019.05.006

[mbo370150-bib-0004] Basit, A. , S. Qadir , S. Qureshi , and S. U. Rehman . 2021. “Cloning and Expression Analysis of Fused Holin–Endolysin From RL Bacteriophage; Exhibits Broad Activity Against Multi Drug Resistant Pathogens.” Enzyme and Microbial Technology 149: 109846. 34311883 10.1016/j.enzmictec.2021.109846

[mbo370150-bib-0005] Blum, M. , A. Andreeva , L. C. Florentino , et al. 2025. “InterPro: The Protein Sequence Classification Resource in 2025.” Nucleic Acids Research 53, no. D1: D444–D456. 39565202 10.1093/nar/gkae1082 PMC11701551

[mbo370150-bib-0006] Boroujeni, M. B. , S. Mohebi , A. Malekian , et al. 2024. “The Therapeutic Effect of Engineered Phage, Derived Protein and Enzymes Against Superbug Bacteria.” Biotechnology and Bioengineering 121, no. 1: 82–99. 10.1002/bit.28581. 37881139

[mbo370150-bib-0007] Broendum, S. S. , A. M. Buckle , and S. McGowan . 2018. “Catalytic Diversity and Cell Wall Binding Repeats in the Phage‐Encoded Endolysins.” Molecular Microbiology 110, no. 6: 879–896. 10.1111/mmi.14134. 30230642

[mbo370150-bib-0008] Cahill, J. , and R. Young . 2019. “Phage Lysis: Multiple Genes for Multiple Barriers.” Advances in Virus Research 103: 33–70. 30635077 10.1016/bs.aivir.2018.09.003 PMC6733033

[mbo370150-bib-0009] Chu, J. J. K. , W. H. Poh , N. T. B. Hasnuddin , et al. 2022. “Novel Phage Lysin Abp013 Against *Acinetobacter baumannii* .” Antibiotics (USSR) 11, no. 2: 169. 10.3390/antibiotics11020169. PMC8868305 35203772

[mbo370150-bib-0010] Danis‐Wlodarczyk, K. M. , D. J. Wozniak , and S. T. Abedon . 2021. “Treating Bacterial Infections With Bacteriophage‐Based Enzybiotics: In Vitro, In Vivo and Clinical Application.” Antibiotics (USSR) 10, no. 12: 1497. 10.3390/antibiotics10121497. PMC8698926 34943709

[mbo370150-bib-0011] Du, Z. , H. Su , W. Wang , et al. 2021. “The trRosetta Server for Fast and Accurate Protein Structure Prediction.” Nature Protocols 16, no. 12: 5634–5651. 34759384 10.1038/s41596-021-00628-9

[mbo370150-bib-0012] Dunne, M. , S. Leicht , B. Krichel , et al. 2016. “Crystal Structure of the CTP1L Endolysin Reveals How Its Activity Is Regulated by a Secondary Translation Product.” Journal of Biological Chemistry 291, no. 10: 4882–4893. 26683375 10.1074/jbc.M115.671172 PMC4777826

[mbo370150-bib-0013] Froger, A. , and J. E. Hall . 2007. “Transformation of Plasmid DNA Into *E. coli* Using the Heat Shock Method.” Journal of Visualized Experiments: JoVE 6: 253. 10.3791/253. PMC2557105 18997900

[mbo370150-bib-0014] Golban, M. , J. Charostad , H. Kazemian , and H. Heidari . 2025. “Phage‐Derived Endolysins Against Resistant *Staphylococcus* spp.: A Review of Features, Antibacterial Activities, and Recent Applications.” Infectious Diseases and Therapy 14, no. 1: 13–57. 10.1007/s40121-024-01069-z. 39549153 PMC11782739

[mbo370150-bib-0015] Gomez‐Raya‐Vilanova, M. V. , K. Leskinen , A. Bhattacharjee , et al. 2022. “The DNA Polymerase of Bacteriophage YerA41 Replicates Its T‐Modified DNA in a Primer‐Independent Manner.” Nucleic Acids Research 50, no. 7: 3985–3997. 10.1093/nar/gkac203. 35357498 PMC9023294

[mbo370150-bib-0016] Gontijo, M. T. P. , G. P. Jorge , and M. Brocchi . 2021. “‘Current Status of Endolysin‐Based Treatments Against Gram‐Negative Bacteria.” Antibiotics (USSR) 10, no. 10: 1143. 10.3390/antibiotics10101143 PMC8532960 34680724

[mbo370150-bib-0017] Guzman, L.‐M. , D. Belin , M. J. Carson , and J. Beckwith . 1995. “Tight Regulation, Modulation, and High‐Level Expression by Vectors Containing the Arabinose PBAD Promoter.” Journal of Bacteriology 177, no. 14: 4121–4130. 7608087 10.1128/jb.177.14.4121-4130.1995 PMC177145

[mbo370150-bib-0018] Hallgren, J. , K. D. Tsirigos , M. D. Pedersen , et al. 2022. “DeepTMHMM Predicts Alpha and Beta Transmembrane Proteins Using Deep Neural Networks.” Preprint, BioRxiv. 10.1101/2022.04.08.487609.

[mbo370150-bib-0019] Han, H. , X. Li , T. Zhang , et al. 2019. “Bioinformatic Analyses of a Potential Salmonella‐virus‐FelixO1 Biocontrol Phage BPS15S6 and the Characterisation and Anti‐Enterobacteriaceae‐Pathogen Activity of Its Endolysin Lys15s6.” Antonie Van Leeuwenhoek 112, no. 11: 1577–1592. 10.1007/s10482-019-01283-7. 31147967

[mbo370150-bib-0020] Jindanuch, M. , A. Chiaha , R. Hughes , et al. 2024. “Investigating Novel *Streptomyces* Bacteriophage Endolysins as Potential Antimicrobial Agents.” Microbiology Spectrum 13, no. 1: e01170‐24. 10.1128/spectrum.01170-24. 39570052 PMC11705968

[mbo370150-bib-0021] Jonas, O. B. , and World Bank Team . 2017. “Drug‐Resistant Infections: A Threat to Our Economic Future.” World Bank Report 2: 1–132.

[mbo370150-bib-0022] Khakhum, N. , U. Yordpratum , A. Boonmee , U. Tattawasart , J. Rodrigues , and R. W. Sermswan . 2016. “Cloning, Expression, and Characterization of a Peptidoglycan Hydrolase From the Burkholderia Pseudomallei Phage ST79.” AMB Express 6, no. 1: 77. 10.1186/s13568-016-0251-7. 27637947 PMC5025407

[mbo370150-bib-0023] Li, X. , C. Zhang , F. Wei , F. Yu , and Z. Zhao . 2021. “‘Bactericidal Activity of a Holin–Endolysin System Derived From Vibrio Alginolyticus Phage HH109.” Microbial Pathogenesis 159: 105135. 34390766 10.1016/j.micpath.2021.105135

[mbo370150-bib-0024] Lu, N. , Y. Sun , Q. Wang , et al. 2020. “Cloning and Characterization of Endolysin and Holin From Streptomyces Avermitilis Bacteriophage phiSASD1 as Potential Novel Antibiotic Candidates.” International Journal of Biological Macromolecules 147: 980–989. 31715241 10.1016/j.ijbiomac.2019.10.065

[mbo370150-bib-0025] Morzywolek, A. , M. Plotka , A. K. Kaczorowska , et al. 2021. “Novel Lytic Enzyme of Prophage Origin From *Clostridium botulinum* E3 Strain Alaska E43 With Bactericidal Activity Against Clostridial Cells.” International Journal of Molecular Sciences 22, no. 17: 9536. 10.3390/ijms22179536. 34502443 PMC8430805

[mbo370150-bib-0026] Naghavi, M. , S. E. Vollset , K. S. Ikuta , et al. 2024. “Global Burden of Bacterial Antimicrobial Resistance 1990–2021: A Systematic Analysis With Forecasts to 2050.” Lancet 404, no. 10459: 1199–1226. 10.1016/S0140-6736(24)01867-1. 39299261 PMC11718157

[mbo370150-bib-0027] Nelson, D. , R. Schuch , P. Chahales , S. Zhu , and V. A. Fischetti . 2006. “PlyC: A Multimeric Bacteriophage Lysin.” Proceedings of the National Academy of Sciences 103, no. 28: 10765–10770. 10.1073/pnas.0604521103. PMC1487170 16818874

[mbo370150-bib-0028] Niaz, H. , M. Skurnik , and F. Adnan . 2025. “Genomic and Proteomic Characterization of Four Novel Schitoviridae Family Phages Targeting Uropathogenic *Escherichia coli* Strain.” Virology Journal 22, no. 1: 83. 10.1186/s12985-025-02691-0. 40119445 PMC11927229

[mbo370150-bib-0029] Nie, T. , F. Meng , F. Lu , et al. 2022. “An Endolysin Salmcide‐p1 From Bacteriophage fmb‐p1 Against Gram‐Negative Bacteria.” Journal of Applied Microbiology 133, no. 3: 1597–1609. 10.1111/jam.15661. 35689810

[mbo370150-bib-0030] Nielsen, H. 2017. “Predicting Secretory Proteins With SignalP.” Protein Function Prediction: Methods in Molecular Biology (Clifton, NJ) 1611: 59–73. 10.1007/978-1-4939-7015-5_6 28451972

[mbo370150-bib-0031] Niu, X. , and M. J. Guiltinan . 1994. “DNA Binding Specificity of the Wheat bZIP Protein EmBP‐1.” Nucleic Acids Research 22, no. 23: 4969–4978. 10.1093/nar/22.23.4969. 7800488 PMC523765

[mbo370150-bib-0032] Pang, T. , C. G. Savva , K. G. Fleming , D. K. Struck , and R. Young . 2009. “Structure of the Lethal Phage Pinhole.” Proceedings of the National Academy of Sciences 106, no. 45: 18966–18971. 10.1073/pnas.0907941106 PMC2776468 19861547

[mbo370150-bib-0033] Park, Y. , J. A. Lim , M. Kong , S. Ryu , and S. Rhee . 2014. “Structure of Bacteriophage SPN1S Endolysin Reveals an Unusual Two‐Module Fold for the Peptidoglycan Lytic and Binding Activity.” Molecular Microbiology 92, no. 2: 316–325. 10.1111/mmi.12555. 24641441

[mbo370150-bib-0034] Proença, D. , C. Velours , C. Leandro , M. Garcia , M. Pimentel , and C. São‐José . 2015. “A Two‐Component, Multimeric Endolysin Encoded by a Single Gene.” Molecular Microbiology 95, no. 5: 739–753. 10.1111/mmi.12857. 25388025

[mbo370150-bib-0035] Rahman, M. U. , W. Wang , Q. Sun , et al. 2021. “Endolysin, a Promising Solution Against Antimicrobial Resistance.” Antibiotics (Basel, Switzerland) 10, no. 11: 1277. 10.3390/antibiotics10111277. 34827215 PMC8614784

[mbo370150-bib-0036] Ramesh, N. , P. Manohar , K. Eniyan , et al. 2022. “A Lysozyme Murein Hydrolase With Broad‐Spectrum Antibacterial Activity From Enterobacter Phage myPSH1140.” Antimicrobial Agents and Chemotherapy 66, no. 9: e0050622. 10.1128/aac.00506-22. 35950843 PMC9487488

[mbo370150-bib-0037] Sabur, A. , A. Khan , B. Borphukan , A. Razzak , M. Salimullah , and M. Khatun . 2025. “The Unique Capability of Endolysin to Tackle Antibiotic Resistance: Cracking the Barrier.” Journal of Xenobiotics 15: 19. 10.3390/jox15010019. 39997362 PMC11856723

[mbo370150-bib-0038] Sambrook, J. , and D. W. Russell . 2006. “Purification of Nucleic Acids by Extraction With Phenol:Chloroform.” Cold Spring Harbor Protocols 2006, no. 1: pdb‐prot4455. 10.1101/pdb.prot4455 22485786

[mbo370150-bib-0039] Seal, B. S. , D. E. Fouts , M. Simmons , et al. 2011. “ *Clostridium perfringens* Bacteriophages ΦCP39O and ΦCP26F: Genomic Organization and Proteomic Analysis of the Virions.” Archives of Virology 156: 25–35. 20963614 10.1007/s00705-010-0812-z PMC4127328

[mbo370150-bib-0040] Skurnik, M. , S. Alkalay‐Oren , M. Boon , et al. 2025. “Phage Therapy.” Nature Reviews Methods Primers 5, no. 1: 9. 10.1038/s43586-024-00377-5.

[mbo370150-bib-0041] Smith, D. L. , D. K. Struck , J. M. Scholtz , and R. Young . 1998. “Purification and Biochemical Characterization of the Lambda Holin.” Journal of Bacteriology 180, no. 9: 2531–2540. 9573208 10.1128/jb.180.9.2531-2540.1998 PMC107198

[mbo370150-bib-0042] Staden, R. 1996. “The Staden Sequence Analysis Package.” Molecular Biotechnology 5, no. 3: 233–241. 8837029 10.1007/BF02900361

[mbo370150-bib-0043] Takáč, M. , A. Witte , and U. Bläsi . 2005. “Functional Analysis of the Lysis Genes of *Staphylococcus aureus* Phage P68 in *Escherichia coli* .” Microbiology 151, no. 7: 2331–2342. 16000723 10.1099/mic.0.27937-0

[mbo370150-bib-0044] Walmagh, M. , B. Boczkowska , B. Grymonprez , Y. Briers , Z. Drulis‐Kawa , and R. Lavigne . 2013. “Characterization of Five Novel Endolysins From Gram‐Negative Infecting Bacteriophages.” Applied Microbiology and Biotechnology 97, no. 10: 4369–4375. 10.1007/s00253-012-4294-7. 22832988

[mbo370150-bib-0045] Wang, I.‐N. , D. L. Smith , and R. Young . 2000. “Holins: The Protein Clocks of Bacteriophage Infections.” Annual Review of Microbiology 54, no. 1: 799–825. 10.1146/annurev.micro.54.1.799 11018145

[mbo370150-bib-0046] Yin, Y. , X. Wang , Z. Mou , et al. 2022. “Characterization and Genome Analysis of *Pseudomonas aeruginosa* Phage vB_PaeP_Lx18 and the Antibacterial Activity of Its Lysozyme.” Archives of Virology 167, no. 9: 1805–1817. 35716268 10.1007/s00705-022-05472-0

[mbo370150-bib-0047] Zhang, M. , Y. Wang , J. Chen , et al. 2022. “Identification and Characterization of a New Type of Holin–Endolysin Lysis Cassette in *Acidovorax oryzae* Phage AP1.” Viruses 14, no. 2: 167. 35215761 10.3390/v14020167 PMC8879335

[mbo370150-bib-0048] Zheng, T. , and C. Zhang . 2024. “Engineering Strategies and Challenges of Endolysin as an Antibacterial Agent Against Gram‐Negative Bacteria.” Microbial Biotechnology 17, no. 4: e14465. 10.1111/1751-7915.14465. 38593316 PMC11003714

