# Supplementary material for: Molecular Cloning and Functional Analysis of Holin and Endolysin From Escherichia Phage UE‐M6 as Potential Antibacterial Agents
Source: Microbiologyopen. 2025 Nov 28;14(6):e70150. doi: 10.1002/mbo3.70150 (PMC12661215; doi:10.1002/mbo3.70150)
Supplement: Supplementary file 1 — Additional file revised. [file MBO3-14-e70150-s001.docx]

**Supplementary file**

**Molecular Cloning and Functional Analysis of Holin and Endolysin from an Escherichia Phage UE-M6 as Potential Antibacterial Agents**

Hira Niaz^1^, Mikael Skurnik*^2^, Fazal Adnan*^1^

1. Atta ur Rahman School of Applied Biosciences (ASAB), National University of Sciences and Technology (NUST), Islamabad 44000, Pakistan

2. Department of Bacteriology and Immunology, Human Microbiome Research Program, Faculty of Medicine, University of Helsinki, Helsinki FI

*Corresponding authors: [adnanfazal@asab.nust.edu.pk](mailto:adnanfazal@asab.nust.edu.pk), [mikael.skurnik@helsinki.fi](mailto:mikael.skurnik@helsinki.fi)

**SUPPLEMENTARY INFORMATION**

**Table S1:** Reagents and kits used in this study

**Table S2**: Bacterial strains used in Antibacterial spectrum

**Figure S1:** Prediction of transmembrane regions in UE-holin using DEEP TMHMM

**Figure S2:**  The domain organization of the UE-lysin: the *N*-terminal domain (red) contains the *N*-acetylmuramidase activity with the location of the catalytic domain (EGGY) indicated, and C- terminal end contains the PG-binding domain (blue).

**Figure S3:** Quality evaluation of predicted (a )UE-holin (b) UE-lysin by Ramachandran plot.

**Figure S4:** Sanger sequencing confirmation of cloned **(A)** UE-lysin and **(B)** UE-holin genes. Sequence alignment with the corresponding reference sequences revealed 100% match, confirming the correct insertion, orientation of both genes. Chromatograms were analysed using the Staden Package programs for the alignment.

**Figure S5:** SDS-PAGE analysis of protein purification samples of UE-lysin expression after 2 washes and 4 elutions from the Ni-NTA columns. **A.** The vector-only control *E. coli* BL21(DE3)/pET28α, **B.** UE-lysin expressed in *E. coli* BL21(DE3). Expressed UE-lysin bands are indicated with white arrows.

**Figure S6:** SDS-PAGE analysis of protein purification samples of UE-holin expression after 2 washes and 4 elutions from the Ni-NTA columns. UE-holin expressed in BL21 strain. Expressed UE-holin bands are indicated with white arrows.

**Figure S7:** Effect of different concentration of EDTA on the growth of host bacterial strain

**Table S1:** Reagents and kits used in this study

| **Sr.no** | **Reagent/test kits** | **Lot number** | **Manufacturer** |
| --- | --- | --- | --- |
|  | Lysogeny broth (LB) | LB996612 | Icon Chemical |
|  | Agar | A10023102 | Bio WORLD |
|  | Agarose | 2963550/2963543 | Thermo Scientific |
|  | Mueller-Hinton agar (MHA) | L20014005LCMB | Bio WORLD |
|  | Chloroform | SZBF0490V | Sigma-Aldrich |
|  | Phenol | P0035PJ1 | DAEJUNG |
|  | Ethylenediaminetetraacetic Acid (EDTA) | SZBC1590V | Sigma-Aldrich |
|  | Sodium Dodecyl Sulfate (SDS) | 80800 | Sigma-Aldrich |
|  | Calcium chloride | C0023QD1 | DAEJUNG |
|  | Imidazole |  | Sigma-Aldrich |
|  | Ammonium persulfate (APS) |  | Merck |
|  | Bromophenol blue |  | Sigma-Aldrich |
|  | Tris Base | Y11705063 | MPI Biomedical |
|  | Isopropyl-β-D thiogalactopyranoside (IPTG) | T15210012 | Bio WORLD |
|  | L-arabinose |  | Sigma-Aldrich |
|  | TEMED | 10201026 | VWR Chemicals |
|  | 30% Acrylamide | MKCL1675 | Sigma-Aldrich |
|  | Kanamycin |  | Sigma-Aldrich |
|  | Ampicillin |  | Sigma-Aldrich |
|  | DNaseI | 3000622 | Thermo Fisher Scientific |
|  | RNaseI | 2991329 | Thermo Fisher Scientific |
|  | HindIII |  | Thermo Fisher Scientific |
|  | BamHI |  | Thermo Fisher Scientific |
|  | Fast digest buffer |  | Thermo Fisher Scientific |
|  | High fidelity DNA polymeras | F530S | Thermo Fisher Scientific |
|  | lysozyme | VG305851 | Thermo Fisher Scientific |
|  | T4 DNA ligase |  | Sigma-Aldrich |
|  | Protease inhibitor tablets |  | Pierce™ Protease Inhibitor mini-Tablets, EDTA-free, Thermo Fisher Scientific |
|  | Pierce Gel Code Blue Stain Reagent |  | Thermo Fisher Scientific |
|  | SanPrep Column PCR Product Purification Kit |  | Sangon Biotech, Shanghai, China |
|  | SanPrep Column Gel Product Purification Kit |  | Sangon Biotech, Shanghai, China |
|  | Plasmid extraction kit (Nucleospin EasyPure Kit) |  | Macherey-Nagel, Düren, Germany) |
|  | Ni-NTA Agarose |  | Sigma-Aldrich |

**Table S2: Bacterial strains used in Antibacterial spectrum**

| **Bacterial species** | **Strain Code** | **Source** | **References** |
| --- | --- | --- | --- |
| ***E. coli*** | PSU-5265 | Human Urine | SAMN27614567 |
|  | UE-87 | Human Urine | AntiBacter Lab, Pakistan |
|  | STEC | Cow faecal | AntiBacter Lab, Pakistan |
|  | CME-5 | Cow milk | SAMN25558087 |
|  | PE-127 | Poultry liver | (1) |
|  | FE-28 | Cow faecal | (2) |
| ***S. aureus*** | G-19 | Cow milk | AntiBacter Lab, Pakistan |
|  | G-21 | Cow milk | AntiBacter Lab, Pakistan |
|  | G-LR | Cow milk | AntiBacter Lab, Pakistan |
| ***Bacillus safensis*** | Bacillus 4B | Soil | (3) |


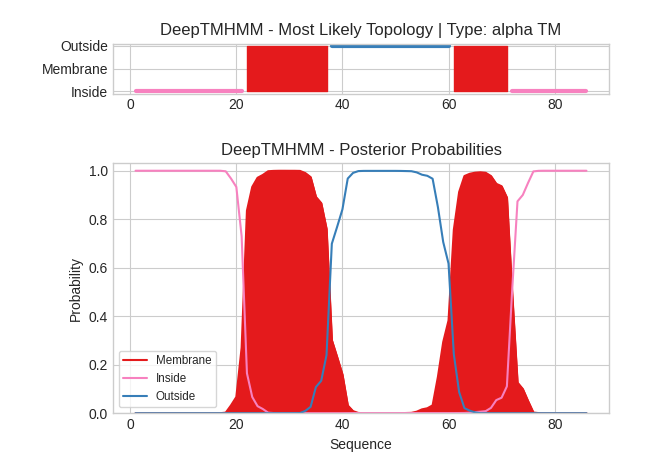


**Figure S1:** Prediction of transmembrane regions in UE-holin using DEEP TMHMM

**
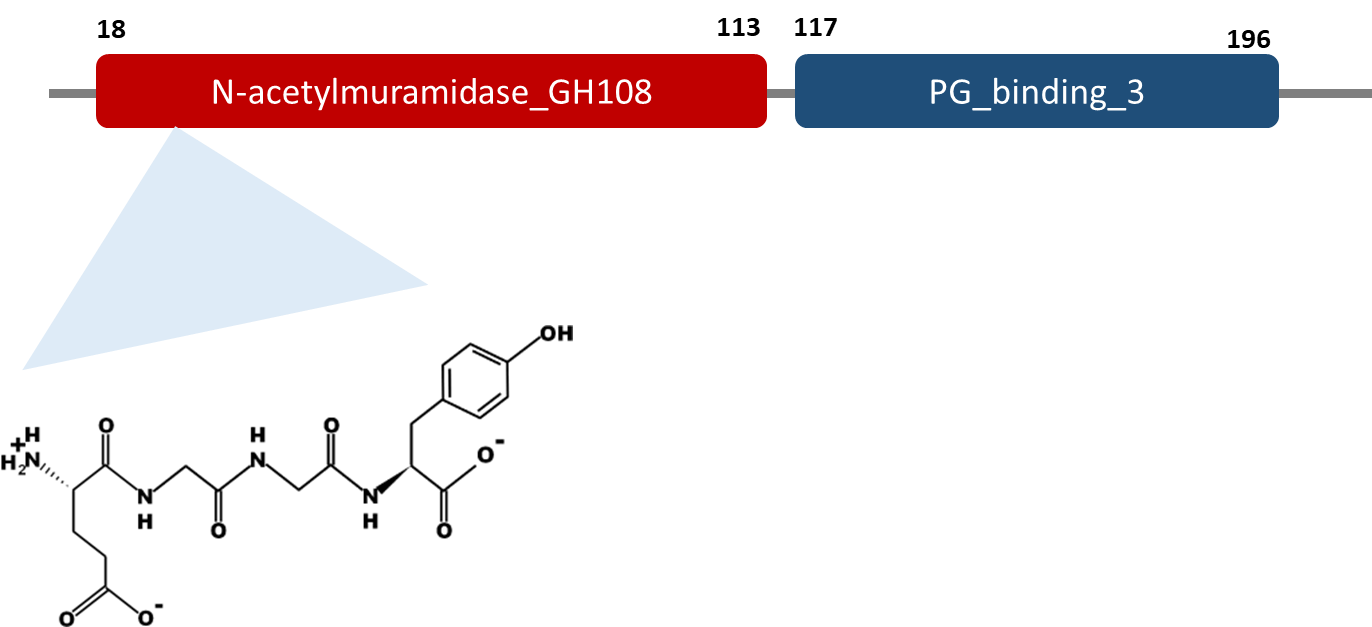
**

**Figure S2:**  The domain organization of the UE-lysin: the *N*-terminal domain (red) contains the *N*-acetylmuramidase activity with the location of the catalytic domain (EGGY) indicated, and C- terminal end contains the PG-binding domain (blue).

**A.**


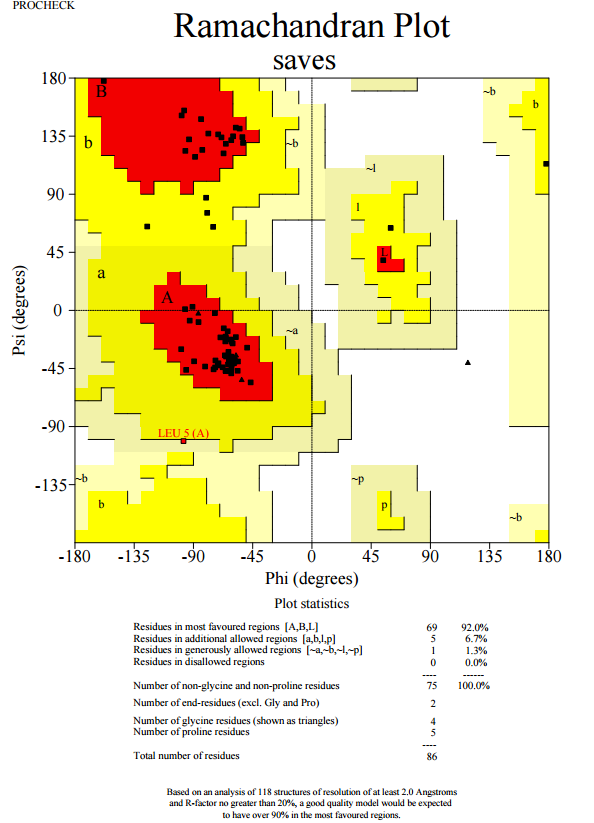


**B.**


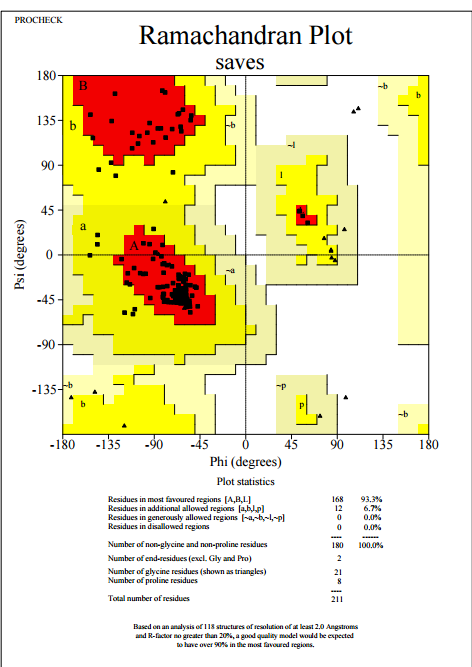


**Figure S3:** Quality evaluation of predicted (a )UE-holin (b) UE-lysin by Ramachandran plot.

**A.**

**
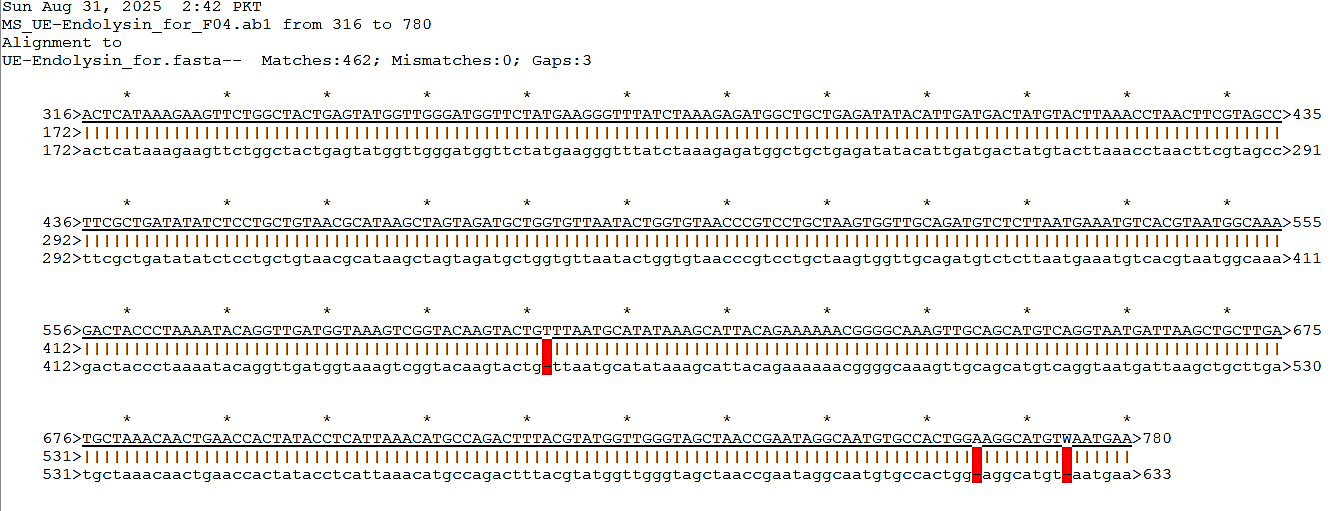
**

**B.**

**
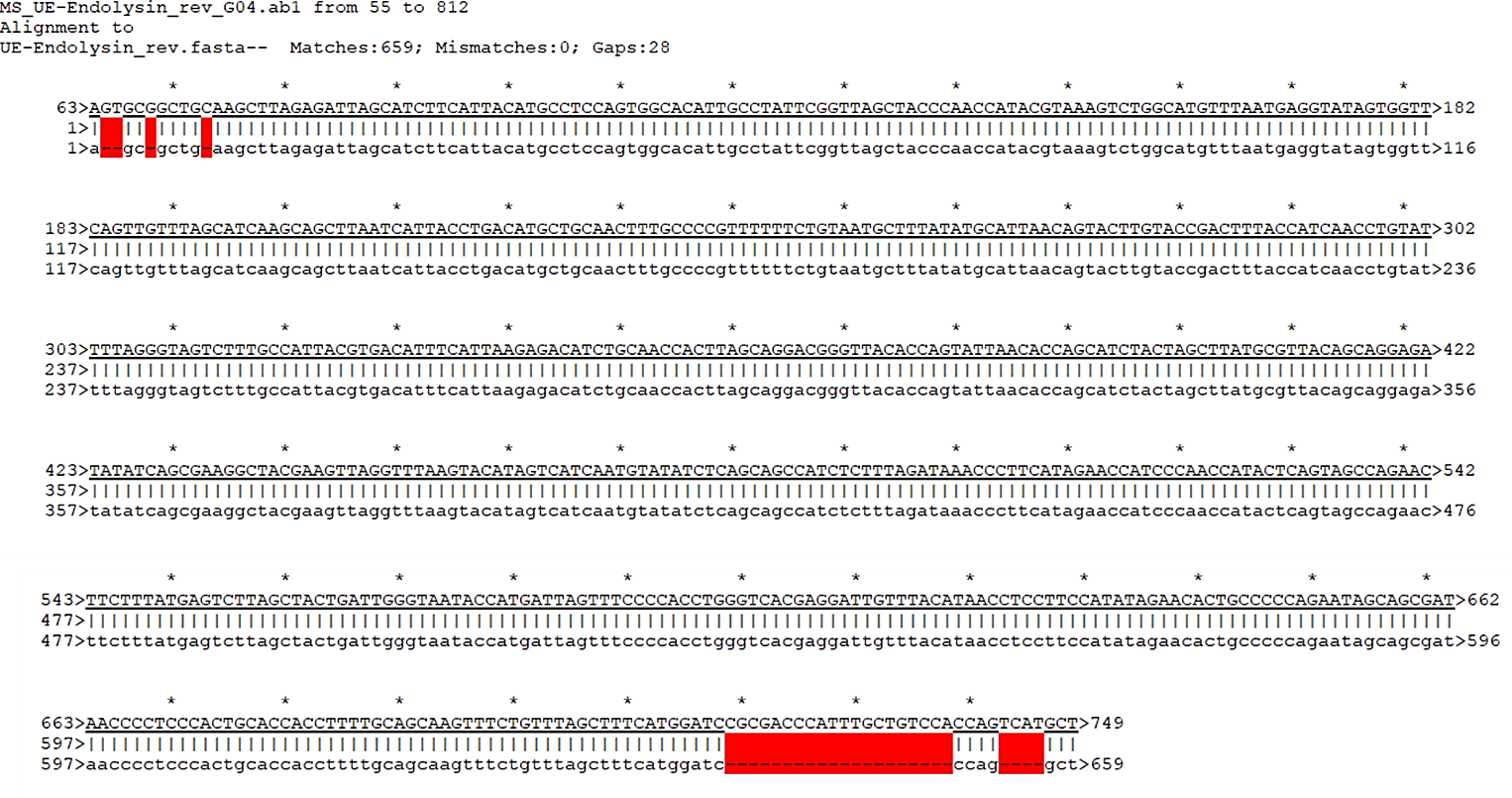
**

**C.**

**
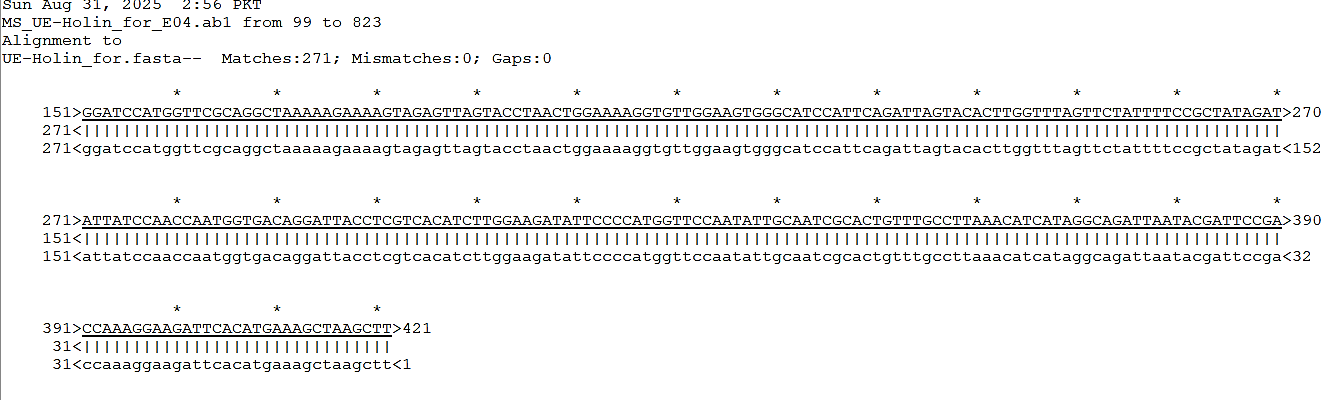
**

**Figure S4:** Sanger sequencing confirmation of cloned **(A)** UE-lysin forward, **(B)** UE-lysin reverse and **(C)** UE-holin forward genes. Sequence alignment with the corresponding reference sequences revealed 100% match, confirming the correct insertion, orientation of both genes. Chromatograms were analyzed using Staden Package programs for the alignment.

A.


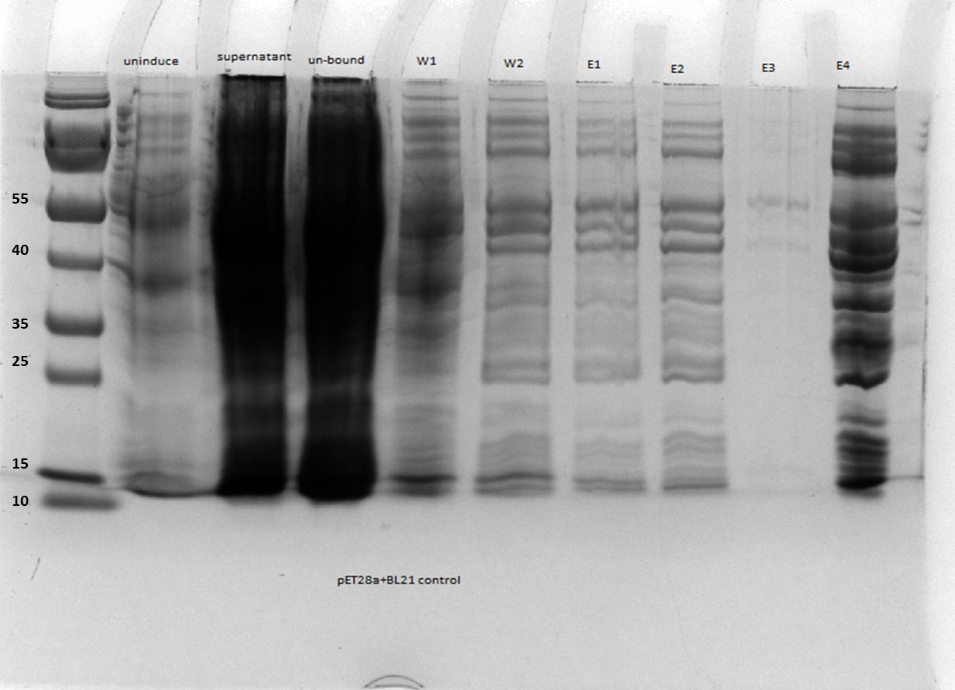


B.


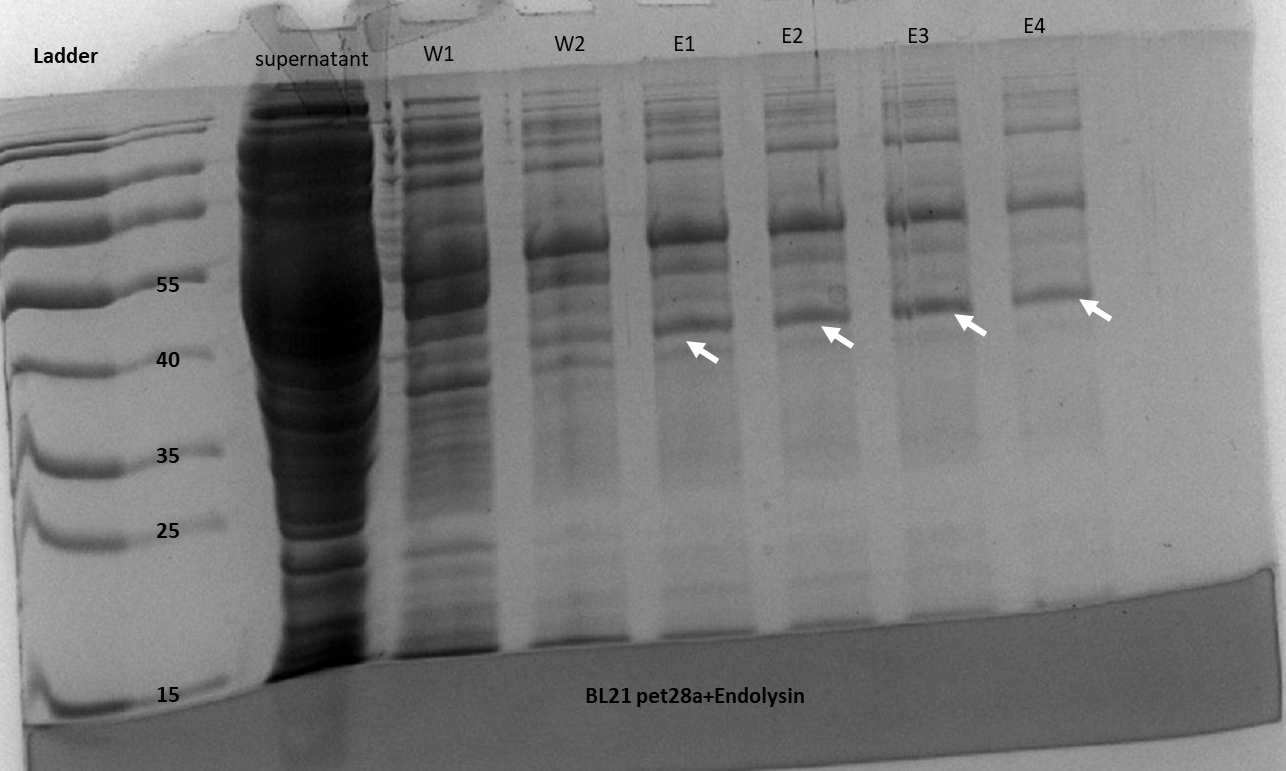


**Figure S5:** SDS-PAGE analysis of protein purification samples of UE-lysin expression after 2 washes and 4 elutions from the Ni-NTA columns. **A.** The vector-only control *E. coli* BL21(DE3)/pET28α, **B.** UE-lysin expressed in *E. coli* BL21(DE3). Expressed UE-lysin bands are indicated with white arrows. W1: primary wash effluent; W2: Secondary wash effluent; E1: primary eluate ; E2: secondary eluate; E3: tertiary eluate; E4: quaternary eluate


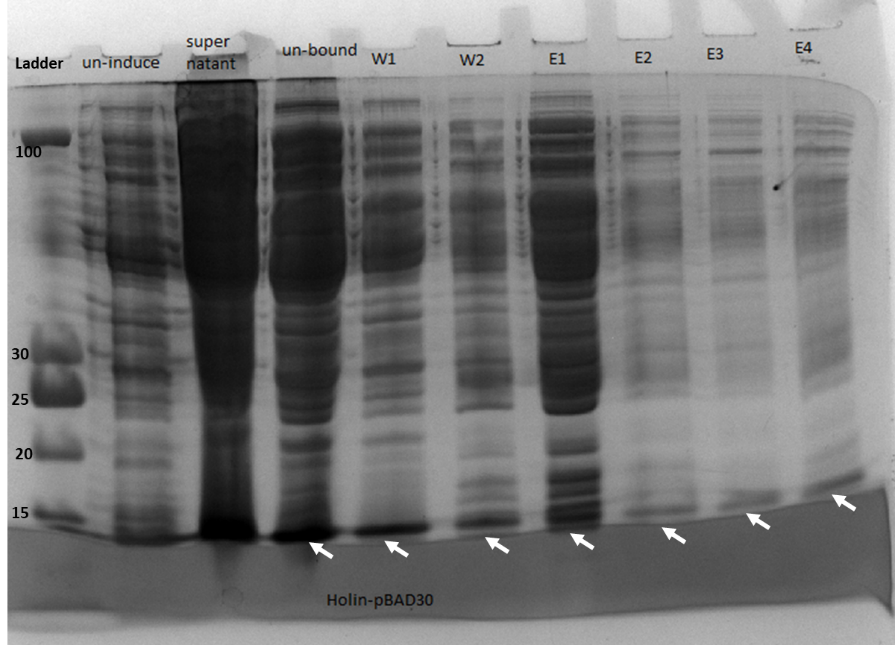


**Figure S6:** SDS-PAGE analysis of protein purification samples of UE-holin expression after 2 washes and 4 elutions from the Ni-NTA columns. UE-holin expressed in BL21 strain. Expressed UE-holin bands are indicated with white arrows. W1: primary wash effluent; W2: Secondary wash effluent; E1: primary eluate ; E2: secondary eluate; E3: tertiary eluate; E4: quaternary eluate


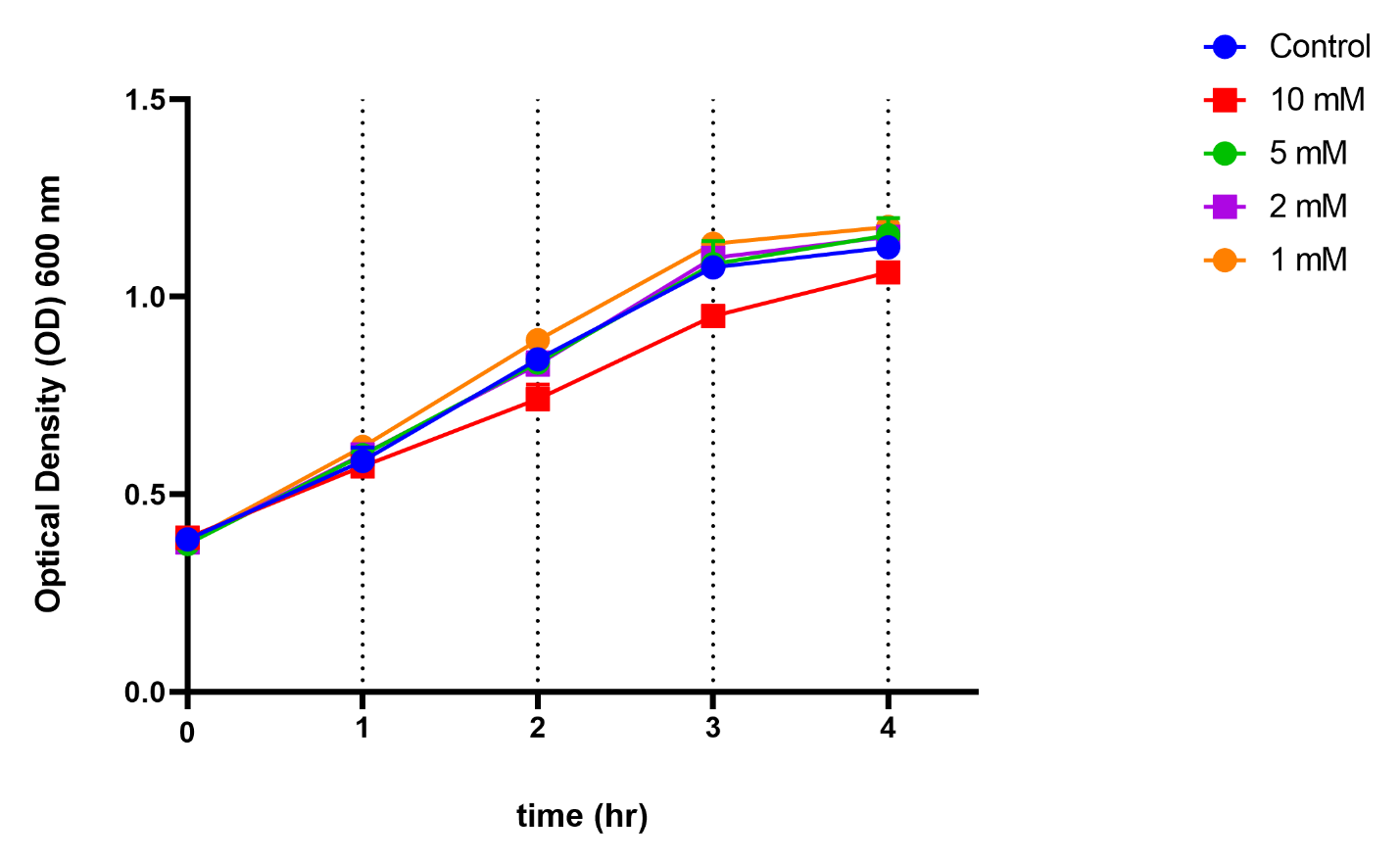


**Figure S7:** effect of different concentration of EDTA on the growth of host bacterial strain

**References:**

1. Jalil A, Masood S, Ain Q, Andleeb S, Dudley EG, Adnan F. High resistance of fluoroquinolone and macrolide reported in avian pathogenic Escherichia coli isolates from the humid subtropical regions of Pakistan. J Glob Antimicrob Resist. 2023;33:5–17.

2. Jalil A, Abbasi A, Ain Q, Hussain Z, Usmani M, Adnan F. Prevalence & risk factors analysis of bovine mastitis in dairy herds of Rawalpindi district, Pakistan; a study to estimate severity & farmers’ awareness about the disease. 2022.

3. Shaheen N, Jalil A, Adnan F, Arsalan Khushnood R. Isolation of alkaliphilic calcifying bacteria and their feasibility for enhanced CaCO3 precipitation in bio‐based cementitious composites. Microb Biotechnol. 2021;14(3):1044–59.
